# Effects of seasonality and previous logging on faecal helminth-microbiota associations in wild lemurs

**DOI:** 10.1038/s41598-020-73827-1

**Published:** 2020-10-08

**Authors:** I. I. de Winter, A. Umanets, G. Gort, W. H. Nieuwland, P. van Hooft, I. M. A. Heitkönig, P. M. Kappeler, H. H. T. Prins, H. Smidt

**Affiliations:** 1 Utrecht University, Padualaan 8, 3584 CH Utrecht, The Netherlands; 2 Laboratory of Microbiology, Wageningen University & Research, Stippeneng 4, 6708 WE Wageningen, The Netherlands; 3 Biometris, Wageningen University & Research, Droevendaalsesteeg 1, 6708 PB Wageningen, The Netherlands; 4 Wildlife Ecology and Conservation Group, Wageningen University & Research, Droevendaalsesteeg 3a, 6708 PB Wageningen, The Netherlands; 5 Behavioral Ecology and Sociobiology Unit, German Primate Center, Kellnerweg 4, 37077 Göttingen, Germany; 6 Animal Sciences Group, Wageningen University & Research, De Elst 1, 6708 WD Wageningen, The Netherlands

**Keywords:** Conservation biology, Ecological genetics, Microbial ecology, Bacteriology, Parasite biology

## Abstract

Gastrointestinal helminth-microbiota associations are shaped by various ecological processes. The effect of the ecological context of the host on the bacterial microbiome and gastrointestinal helminth parasites has been tested in a number of ecosystems and experimentally. This study takes the important step to look at these two groups at the same time and to start to examine how these communities interact in a changing host environment. Fresh faecal samples (N = 335) from eight wild *Eulemur* populations were collected over 2 years across Madagascar. We used 16S ribosomal RNA gene sequencing to characterise the bacterial microbiota composition, and faecal flotation to isolate and morphologically identify nematode eggs. Infections with nematodes of the genera *Callistoura* and *Lemuricola* occurred in all lemur populations. Seasonality significantly contributed to the observed variation in microbiota composition, especially in the dry deciduous forest. Microbial richness and *Lemuricola* spp. infection prevalence were highest in a previously intensely logged site, whereas *Callistoura* spp. showed no such pattern. In addition, we observed significant correlations between gastrointestinal parasites and bacterial microbiota composition in these lemurs, with 0.4–0.7% of the variation in faecal bacterial microbiota composition being explained by helminth infections. With this study, we show effects of environmental conditions on gastrointestinal nematodes and bacterial interactions in wild lemurs and believe it is essential to consider the potential role of microbiome-parasite associations on the hosts’ GI stability, health, and survival.

## Introduction

The gastrointestinal (GI) microbiota plays a vital role in the physiology, health, and nutrition of its host^1^. Next to the microbiota, GI macroparasites, including protozoa and nematodes, can be present within a host’s digestive tract. Parasitism can impact the host’s health, behaviour, and survival, thereby influencing evolutionary processes and population dynamics^2^. Besides, parasites are known to affect the host’s reproduction directly through pathologic effects and mate choice as well as indirectly by impaired nutrition and energy deficits^3^. Researchers suggest a strong connection between specific parasite species and GI bacteria. For example, GI microbiota can prevent gut colonisation by pathogenic microorganisms^4^ and researchers detected a link between the microbiome and simian immunodeficiency virus in wild primates^5^. A stable and diverse GI microbiota composition is crucial for mammalian health^6, 7^, and defining the mechanisms influencing its composition and diversity is considered important^8^.

Faecal bacterial GI microbiota and macroparasites living at internal body surfaces and the lumen of the GI tract are part of an animals’ microbiome and are involved in key host functions^9^. The coinfection of a host by multiple microorganisms has important epidemiological and clinical implications, which has been recognised for decades across multiple animal taxa^10^. As studying wild populations under natural conditions is rather complex, most studies on the determinants of the GI microbiota composition and parasite prevalence either comprise laboratory or clinical studies that focus on a single host species or infection with a single parasite species^11, 12^. Our work is building on this extensive body of literature on GI bacteria and helminths and their interaction to increase our understanding of ecological processes that shape composition and functionality of GI microbiota and parasites in wild populations^12^.

The composition of the GI microbiota is known to be shaped by multiple factors, including host genetics, evolutionary history, physiology, sex, and age^13, 14^. Several recent studies showed that the microbial composition can remain stable over the host’s lifespan^15, 16^. However, other studies found that extrinsic factors, including diet composition^17–19^, pathogens^20^, seasonality^21^, habitat degradation^22^, and geographical differences^23^ influence GI microbiota. For example, it has been shown that the microbial composition in black howler monkeys (*Alouatta pigra*) differs across seasons and is correlated with diet^24^. Also, the distribution of parasite infections in wild host populations is influenced by a number of factors, including host susceptibility and exposure^25^. Many helminths can spread through a variety of transmission modes, including the faecal-oral route, which involves ingestion of contaminated soil or food^26^. The nematodes that are the focus of the present study spend part of their life cycle outside of the host and are therefore exposed to environmental conditions that shape temporal variations in parasite infections. Climatic seasonality has been identified as an important driver of this temporal variation in several wild primate species^27, 28^. However, studies investigating these links have yielded different outcomes^24, 29, 30^. It has also been shown that some nematodes have an accelerated development and increased reproduction and survival rates in wetter and warmer conditions^28, 31^, and desiccate more frequently under dry circumstances^27^. Several studies found GI parasite richness, prevalence, and abundance to be higher in the warm wet season, compared to the cold dry season, e.g., in lemurs^32–34^, chimpanzees^27^, as well as howler and spider monkeys (*Ateles geoffroyi*)^35^. However, some helminth species (e.g., *Enterobius* spp.) seem to prefer relatively low temperatures^36^. Although the underlying processes remain unclear^37^, these examples show that environmental factors can influence the microbial composition and parasite prevalence^14, 21^, and require further study in wild mammals.

In addition to environmental factors, the impact of anthropogenic forest disturbance, including logging, on health and pathogens in both wildlife and humans may be far-reaching (Keele et al.^38^). Anthropogenic forest disturbance may lead to changes in host population densities and interaction patterns of wildlife with humans, domestic animals, and other wildlife species^31, 39^. Such disturbances can thereby enforce changes in the GI microbiota composition and parasite infections^14, 22, 40^. Microbiota diversity can be reduced in degraded areas, as has been shown in howler monkeys, red colobus monkeys (*Procolobus gordonorum*), and other primate species^14, 22, 41^. Furthermore, increased parasite prevalence, virulence, and transmission rates were found in such disturbed forests^40, 42, 43^. Although the exact mechanisms influencing the microbial composition and parasite infections in disturbed forests is still unknown, nutritional stress is considered important^44^. Nutritional stress can alter the microbiome and lower an animal’s immune status, resulting in a higher susceptibility to parasites^45^. Forest disturbance can also directly influence parasites that spend part of their life cycle outside of the host, as changes in forest structure lead to differences in light exposure, temperature, and humidity^46^. This integrated study on forest disturbance effects on both the parasites and the microbiome further explores the parasite and microbiome ecology in wild primates living in natural versus human-modified forests.

Microbiota and parasites co-inhabit the GI-tract and have evolved in close association, suggesting that they have the potential to influence each other^47^. Research on this interplay between host, parasites, and the microbiome has increased over the last decade^48^, and recent studies in humans showed associations between nematode infections and changes in the GI microbiota structure^49–51^. However, this observation is not consistent across human populations^52, 53^. Another study experimentally demonstrated that the gut bacterial composition in mice (*Mus musculus*) can change when exposed to a GI parasite (*Trichuris muris*)^54^. Associations between specific bacteria and the abundance of enteric nematodes were also found in wild wood mice (*Apodemus sylvaticus*)^21^. Most of these studies mentioned above focussed on mice, pigs (*Sus scrofa*), or humans. However, recent studies have begun to address the interaction between the microbiome and parasites in primates^55^, and we aim to contribute with this study more comparative data on the interactive effect of parasite infections and microbiota composition of wild lemurs.

Specifically, we aim to assess the effects of seasonality (i.e., dry versus wet season), and forest disturbance on the interaction between GI parasites and bacterial microbiota composition in two lemur species. Recently, the microbial composition of lemurs has been studied in captive lemurs^15^, in two sympatric wild lemur species^56^, and in wild sifakas^57^. However, the processes leading to the natural variation of faecal microbiota in wild lemurs, and how its variation is influenced by environmental conditions, need further study. Furthermore, only a few studies to date have used a metataxonomic 16S ribosomal RNA (rRNA) gene-targeted approach to address the association and interactive effects between parasites and the microbiome^47, 50, 52–54, 58–60^. In the present study, we focus on four congeneric lemur species at eight geographic locations: *Eulemur rufifrons, E. fulvus, E. macaco*, and *E. rubriventer*. The substantial heterogeneity in lemur habitats across Madagascar is created by an interaction of the east–west and north–south rainfall gradient^61^. The four lemur species belong to the genus *Eulemur* and are morphologically alike^62^, are present in the distinct geographic regions of Madagascar, and inhabit both large intact forests and forests that have experienced past logging^63^.

Given the major role of environmental factors in shaping seasonal variation in microbial community structure and parasite infections, we expected that (1) lemurs inhabiting the dry deciduous forests of western Madagascar with strong seasonal variation in rainfall and temperature show larger seasonal contrasts in both parasite infections and microbial composition compared to lemurs in the rainforests of eastern Madagascar with less seasonal variation. We further expected (2) that the microbiota composition is altered and parasite infection prevalence is increased in lemurs whose habitat is restricted to previously logged rainforests compared to lemurs living in less disturbed forests. Lastly, we explored (3) correlations between GI microbiota and natural parasite infections. Hence, in this study, we determine how the GI microbiota and parasite infections vary with their geographic distribution spatially in wild lemurs along with seasonal variation and past logging. In addition, we explore the interactive effects between the parasites and microbiota present.

## Results

### Seasonality

**Figure 1:**
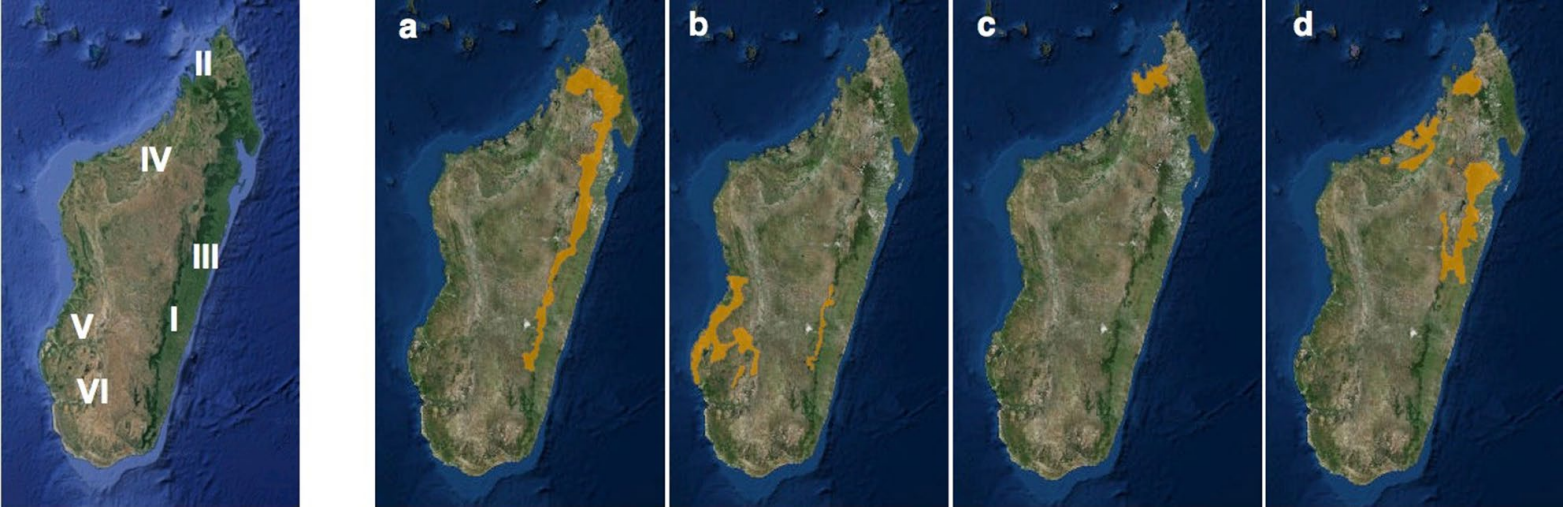
Study sites and the geographic ranges of the different *Eulemur* species (Google Maps, 2015). Left: Map of Madagascar with the study sites Ranomafana NP (I), Nosy Be, Nosy Komba, and Nosy Tanikely (II), Andasibe NP (III), Ankarafantsika NP (IV), Kirindy Forest Reserve (V), Zombitse NP (VI). Right: the geographic ranges of (**a**) *E. rubriventer*, (**b**) *E. rufifrons,* (**c**) *E. macaco,* d) *E. fulvus*^65^. Downloaded from The IUCN Red List of Threatened Species. Version 2016–3. www.iucnredlist.org on 12 February 2017. (**a**) *Eulemur rubriventer*—IUCN (International Union for Conservation of Nature) 2014. Eulemur rubriventer. The IUCN Red List of Threatened Species. Version 2016–3; (**b**) *Eulemur rufifrons*—IUCN (International Union for Conservation of Nature) 2014. Eulemur rufifrons. The IUCN Red List of Threatened Species. Version 2016–3; (**c**) *Eulemur macaco*—IUCN (International Union for Conservation of Nature) 2014. Eulemur macaco. The IUCN Red List of Threatened Species. Version 2016–3; (**d**) *Eulemur fulvus*—IUCN (International Union for Conservation of Nature) 2014. Eulemur fulvus. The IUCN Red List of Threatened Species. Version 2016–3.

**Figure 2:**
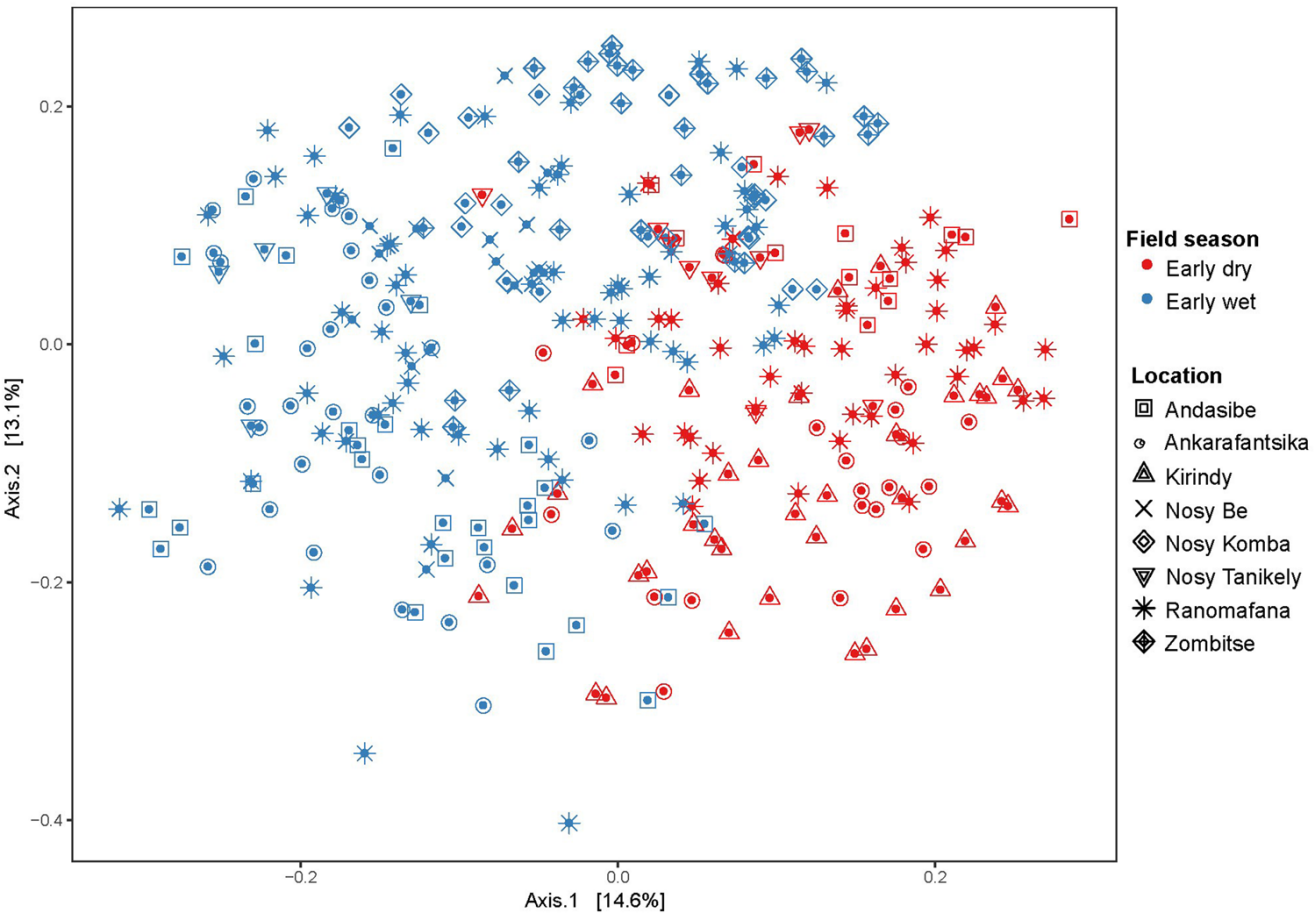
Lemur faecal microbiota composition across seasons and locations. Ordination of faecal microbial composition in multiple lemur populations across Madagascar samples in different seasons (early dry and early wet) and locations. This figure shows the results of a principal coordinate analysis (PCoA) based on the weighted UniFrac distance matrix, grouping strength of samples by season- R^2^ = 0.09 (Adonis; P = 0.001).

**Figure 3:**
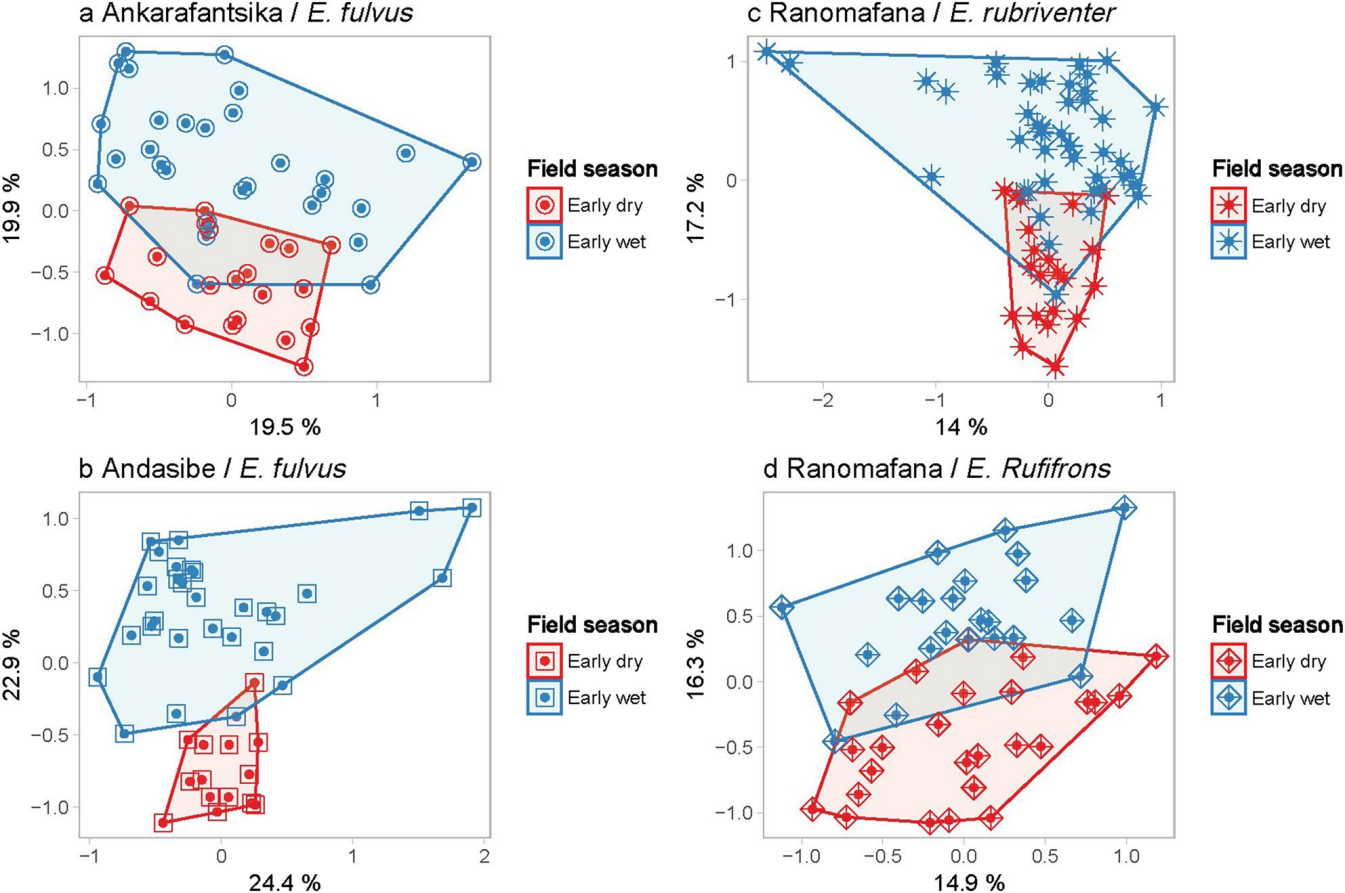
Lemur faecal microbiota composition across seasons and locations. dbRDA Analyses of the abundance-weighted phylogenetic composition at OTU level of individual lemurs across seasons (early dry and early wet) in different geographic areas visualised in ordination. Faecal microbiota significantly cluster by season. Results are given for the percentage of variation explained by the sum of the first two canonical axes, percentage explained by season with corresponding P-value. (**a**) *Eulemur fulvus* in Ankarafantsika National Park, (39.8%, 16.9%, P = 0.001). (**b**) *Eulemur fulvus* in Andasibe (46.5%, 20.2%, P = 0.001. (**c**) *Eulemur rufifrons* in Ranomafana NP (31.1%, 13.5%, P = 0.001). d) *Eulemur rubriventer*, Ranomafana NP (31.3%, 12.5%, P = 0.001).

We found a clear separation of samples by season in the bacterial microbiota composition of multiple lemur populations sampled across Madagascar (Fig. 1, Table 1), using principal coordinate analysis (PCoA) based on the weighted UniFrac distance matrix (early wet season N = 128, early dry season N = 196, R^2^ = 0.08, Adonis; P = 0.0001, Fig. 2). Distance-based redundancy analysis (dbRDA) identified the area of sample collection as the most influential variable followed by season when considering all samples as a single dataset. We observed an increase in the percentage of explained variance in microbiota composition by seasonality when we focused on samples collected within one area and one lemur species (Fig. 3). Specifically, for *E. fulvus* populations from Ankarafantsika NP and Andasibe NP, and *E. rubriventer* and *E. rufifrons* populations from Ranomafana NP, the percentage of variation in microbiota composition explained by season increased from 5.7% for the entire dataset to 16.9%, 20.2%, 12.5% and 13.5%, respectively (Fig. 3a–d). Therefore, these populations harboured a different microbial composition in the early dry season compared to the early wet season. With regards to alpha diversity, the *E. fulvus* population in Ankarafantsika showed a significantly higher mean phylogenetic diversity (PD index, P < 0.001) in the early dry season (N = 21) compared to the early wet season (N = 29). No statistically significant differences in alpha diversity were observed for other subsets of samples as defined by the area of habitation and lemur species, as we showed in a previous study^64^ (Supplementary Material S1, Table S1).

**Table 1:** Lemur study sites in Madagascar.

Study site	GPS coordinates (S, E)	Area (km^2^)	Annual rainfall (mm)	Mean temperature (annual range, °C)	Altitude (range, m)	Lemur species	Samples (N)
Ranomafana NP	21.27, 47.33	435	3000	11–25	500–1500	*E. rufifrons* *E. rubriventer*	48 68
Nosy Be	13.33, 47.25	252	2250	15–35	0–430	*E. macaco*	18
Nosy Komba	13.47, 48.35	25	2250	15–35	0–620	*E. macaco*	23
Nosy Tanikely	13.47, 48.23	0.3	2250	15–35	0–47	*E. fulvus*	17
Andasibe NP, Mitsinjo	18.92, 48.42	155	1680	10–27	900–1060	*E. fulvus*	43
Ankarafantsika NP	16.25, 46.80	1 350	1300	17–28	80–330	*E. fulvus*	50
Kirindy	20.07, 44.67	722	767	19–31	20–90	*E. rufifrons*	40
Zombitse NP	22.87, 44.68	200	740	14–30	485–825	*E. rufifrons*	31

**Figure 4:**
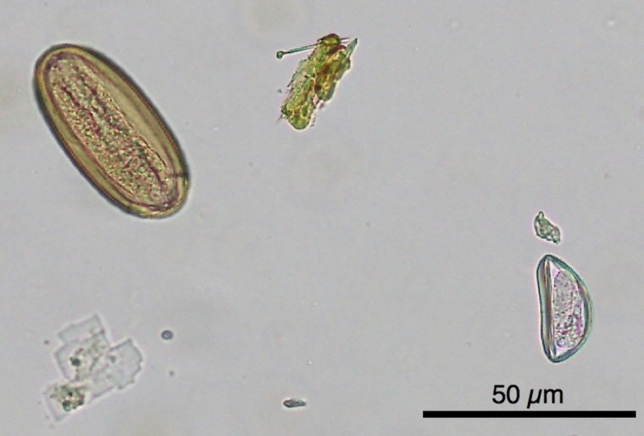
Detected parasite species. *Callistoura* sp. egg (left) and *Lemuricola* sp. egg (right), isolated from a faecal sample of *Eulemur rufifrons,* magnification 200x (*picture taken by IdW*).

Based on morphological analyses, nematode species of two genera, *Callistoura* and *Lemuricola,* were present in the GI tract of nearly all *Eulemur* individuals from eight geographically distinct populations (Fig. 4, Supplementary Material S1). Of all the sampled lemurs (N = 335), we detected eggs of *Callistoura* spp. in 188 (56.1%), eggs of *Lemuricola* spp. in 17 (5.1%), eggs of both parasite species in 34 (10.1%), and no eggs in 96 (28.7%) of the lemurs (Table 2). The observed co-occurrence (10.1%) is very close to the expected co-occurrence for independent infections (67.5% × 15.1% = 9.9%), suggesting that infections with both *Callistoura* and *Lemuricola* spp. occur independently, and therefore, coinfection appears to be independent.

**Table 2:** *Callistoura* and *Lemuricola* spp. prevalence (0–1) in lemur populations at different areas across Madagascar.

	No nematodes	*Lemuricola* spp.	*Callistoura* spp.	Both *Callistoura* and *Lemuricola* spp.	Total (N)
Andasibe NP	0.37	0.02	0.56	0.05	43
Ankarafantsika NP	0.70	0.06	0.20	0.04	50
Kirindy Forest	0.05	0.05	0.73	0.16	37
Nosy Be	0.22	0.11	0.61	0.06	18
Nosy Komba	0.13	0.13	0.48	0.26	23
Nosy Tanikely	0.29	0.06	0.53	0.12	17
Ranomafana NP	0.25	0.03	0.62	0.10	116
Zombitse	0.07	0.03	0.77	0.13	31
Total	0.29	0.05	0.56	0.10	335

In the analysis of *Callistoura* spp. prevalence across seasons and locations using GLMMs, we did not find alarming problems regarding model diagnostics (Supplementary Material S1). A highly significant full model was found (LRT; P < 0.001, Supplementary Material S1; Table S1; model SC1 vs SC2). This result was solely attributed to the random part of the model (SC1 vs SC3), with a larger part being explained by variation among social groups, representing binomial overdispersion (SC1 vs SC5, P = 0.0004), compared to variation among sites (SC1 vs SC6; P = 0.021). We found no significant effect of species, location, season, and the interaction between location and season (SC1 vs SC4; P = 0.20). The hypothesised interaction of location and season was not significantly different from zero (SC1 vs SC7; P = 0.32).

Also, in the analysis of *Lemuricola* spp. prevalence across seasons and locations using GLMMs, no alarming problems regarding model diagnostics were found (Supplementary Material S1). The full model explained a significant amount of variation (Supplementary Material S1; Table S1; model SL1 vs SL2; P = 0.012). We did not find significant variation due to random effects for sites or social groups, and hence no binomial overdispersion (SL1 vs SL3; P = 0.50), but the fixed part of the model was significant (SL1 vs SL4; P = 0.026). The hypothesised interaction of location and season was not found (SL1 vs SL6; P = 0.84), but the location main effect was significant (SL1 vs SL7; P = 0.032). The *Lemuricola* spp. prevalence was estimated as 25% (95% Confidence Interval (CI): 16%-37%) in the dry Western areas compared to 10% (5%-20%) in the wet Eastern areas.

No difference in infection prevalence of *Callistoura* spp. between animals with and without *Lemuricola* spp. infection was found (LRT; P = 0.37). Overall, we found that 188 out of 284 lemurs without *Lemuricola* spp. were infected with *Callistoura* spp. (66%), and 34 out of 51 *Lemuricola* spp. infected animals were infected with *Callistoura* spp. (67%).

### Disturbance

**Figure 5:**
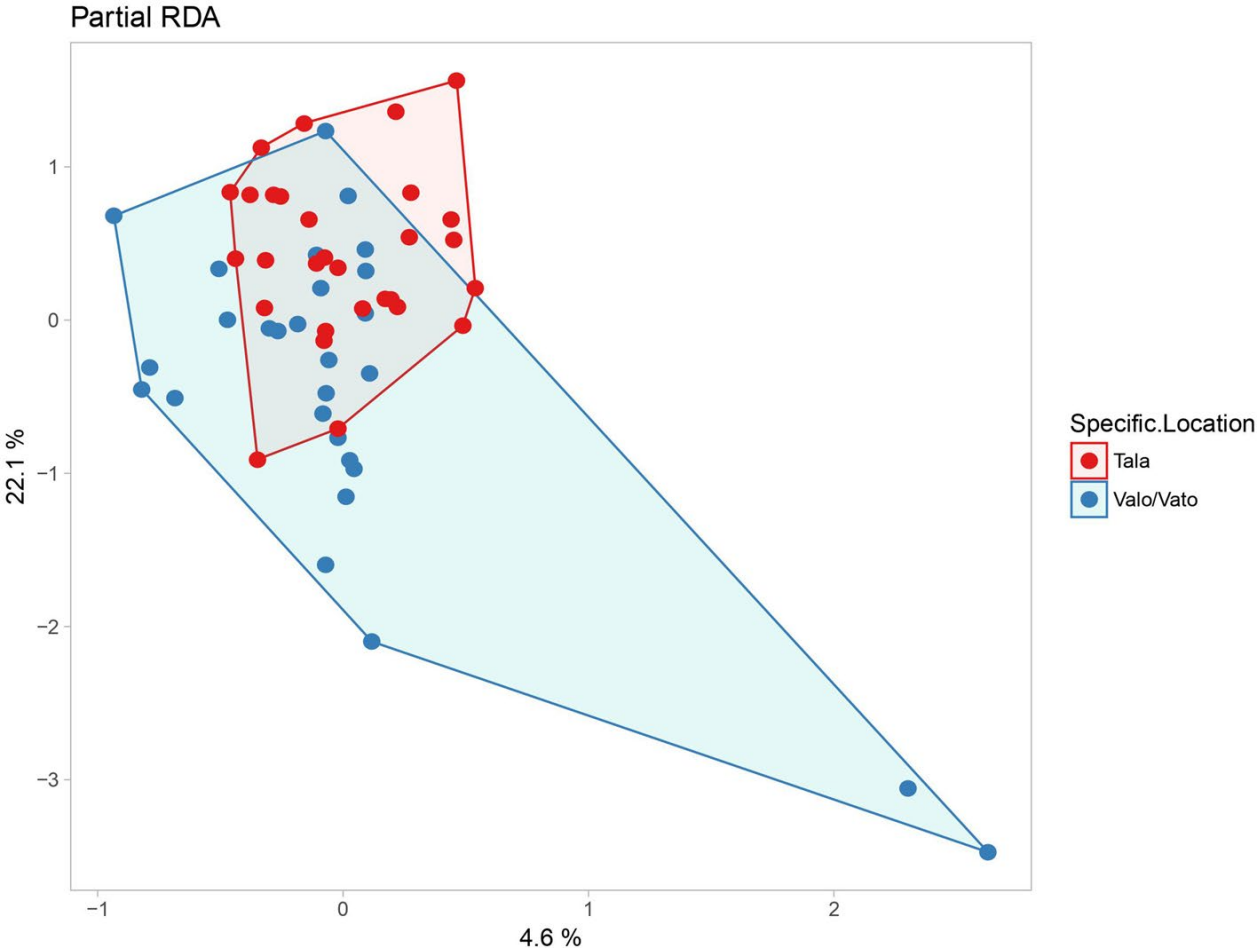
Faecal microbiota composition in disturbed and less disturbed sites. Ordination (RDA) of the microbial composition (OTU) across sites with a different disturbance history (disturbed vs. less disturbed) for *Eulemur rubriventer* and *E. rufifrons* in Ranomafana National Park, Madagascar. Cumulative variation explained by the first two axes was 26.7% and the sampling location accounted for 3.8% of the total variation (P = 0.002).

A possible association between forest disturbance and parasite infection and faecal bacterial microbiota composition was examined in lemurs from Ranomafana NP (Fig. 5). Bacterial richness was significantly higher in the previously logged site (Talatakely, N = 29), compared to the less disturbed site (Vatoharanana/Valohoaka N = 27) (PD index = 7.3 ± 1.1 vs 5.8 ± 1.7, P = 0.001). The dbRDA also showed that the microbial composition was grouped according to sites with a different disturbance history (P = 0.004, Fig. 5).

**Figure 6:**
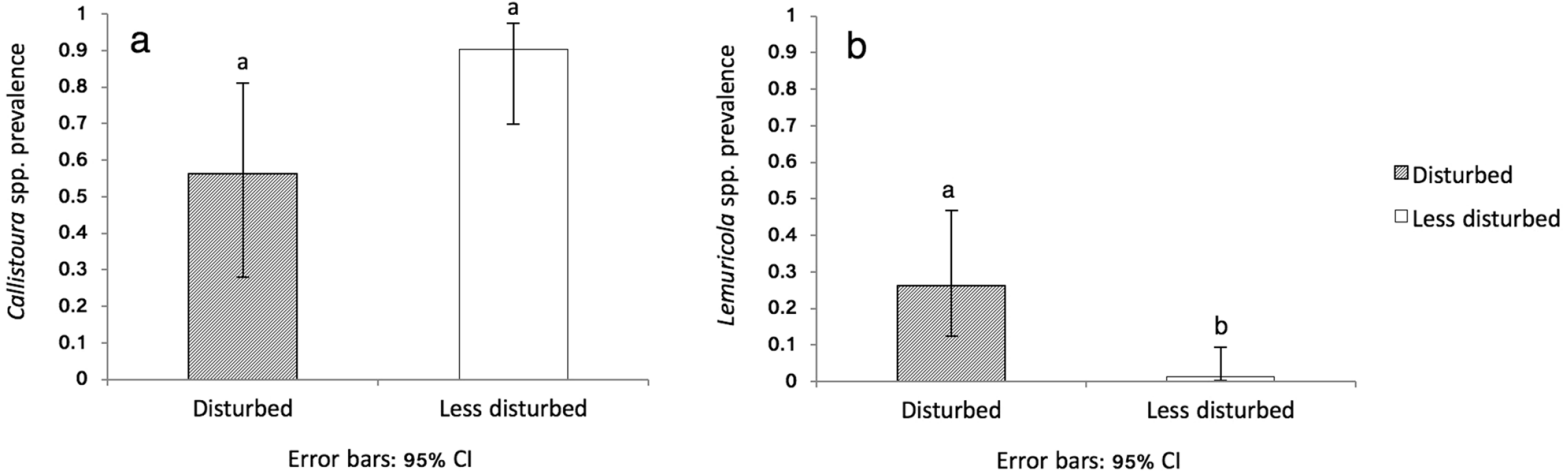
Parasite prevalence with disturbance. (**a**) *Callistoura* spp. prevalence, (**b**) *Lemuricola* spp. prevalence in *Eulemur rufifrons* and *E. rubriventer* populations in a previously disturbed and less disturbed site in Ranomafana NP, Madagascar. Mean with 95% confidence intervals and the letter coding above the bars indicate whether groups are significantly different.

In the analysis of prevalence of *Callistoura* spp. after model checking (Supplementary Material S1), we did not find an overall significant effect of species, disturbance, season or their interaction (Supplementary Material S1; Table S1; model DC1 vs DC2, P = 0.185). When we focus on the specific hypothesis on disturbance, though, a significantly different prevalence between the two subsites was found (model DC1 vs DC3, P = 0.042). The prevalence of *Callistoura* spp. in the non-disturbed subsite was 85% (95% CI 72–93%) and in the disturbed subsite 53% (36–70%, Fig. 6).

In the analysis of the prevalence of *Lemuricola* spp., the omnibus test showed highly significant effects of species (Supplementary Material S1; Table S1; model DL1 vs DL2; P < 0.001), which could not be traced back to differences between subsites with different disturbance histories (model DL1 vs DL4; P < 0.001). The prevalence in the less disturbed compared to the previously logged subsite was estimated as 1.2% (95% CI 02–09%) compared to 26% (15–47%) in the less disturbed subsite. The infection rates of *Callistoura* spp. showed considerable extrabinomial variation, but the infection rates of *Lemuricola* spp. did not.

### Microbiota and parasites

Constrained ordination (dbRDA) showed that the prevalence of *Callistoura* spp. accounted for 0.4% (P = 0.024) of the variation in microbiota composition found among all samples with available microbial and parasite infection data (N = 324), controlling for host species and habitation^64^. However, we did not observe significant (P = 0.49) influence of *Lemuricola* spp. prevalence on microbiota composition. When samples from different seasons were analysed separately, we observed an increase of the relative weight of explained variations to 0.7% with maintained, albeit decreased (due to smaller sample size) significance (P = 0.05) in samples collected during the early-wet season, and no significant influence during the early-dry season.

When focussing on lemurs of one species from the same area and season, we could not find statistically significant correlations with *Callistoura spp.* prevalence. However, among the *E. rubriventer* population in Ranomafana NP in the early dry season, microbiota composition showed a nearly significant correlation with *Lemuricola* spp. prevalence (P = 0.055) with 9.2% of variation explained by this factor. Interestingly, a clear separation of samples could be observed in the corresponding dbRDA plots (Figs. S1, S2), albeit without statistical support (all P > 0.05), potentially due to the relatively low and unequal number of samples per group.

## Discussion

We assessed the influence of environmental conditions on the faecal bacterial microbiota composition and parasite infections as well as the correlation between GI microbiota and parasites in wild lemurs. The two helminth genera *Callistoura*^66^ and *Lemuricola*^67^ were the only two nematode genera detected in all *Eulemur* populations. These microphagous pinworms belong to the family Oxyuridae and are directly transmitted^68^. They colonise distinct parts of the GI tract of their hosts: *Callistoura* spp. lives in the ileum and colon and *Lemuricola* spp. in the caecum and colon^68^. These parasite species were also found in most other lemur genera^67, 68^, including other species from the genus *Eulemur*, i.e. in *E. flavifrons*^69^, *E. macaco*^70^, *E. fulvu*^71^, and *E. albifrons*^72^. So, these nematode genera have an extensive distribution throughout Madagascar and do not show apparent specificity to a particular kind of lemur host^68^.

We hypothesised that lemurs inhabiting dry deciduous forests, with substantial seasonal variation in rainfall and temperature, would show larger seasonal contrasts in both parasite infections and microbial composition compared to lemurs in eastern rainforests with relatively low seasonal variation. Nevertheless, we found a strong seasonal contrast in the microbial composition at the Organisational Taxonomic Unit (OTU) level across all lemur populations. Across Madagascar, lemurs are exposed to seasonality and have been observed to change their diet accordingly^63^. Diet was found to be an important driver of the GI microbial composition in many human studies (e.g.^17^). Although humans are assumed to have a stable microbiota over longer periods (> 10 days)^73^, dietary changes can alter the relative abundance of specific members of the microbiota within 24 h^19^. With respect to wildlife, e.g., wood mice (*Apodemus sylvaticus*) were shown to exhibit seasonal shifts in gut microbiota structure that coincide with their annual dietary changes^21^. Also, in wild Mexican black howler monkeys, temporal changes in the relative abundance of gut bacteria were strongly correlated with dietary variations^24^. Another study on *Eulemurs* show that difference in diet in geographically separated population strongly influence intestinal microbiota^64^. Hence, seasonal diet shifts are likely to explain most of the variation in microbiota in lemurs across seasons.

In addition, the microbial alpha richness from lemurs in Ankarafantsika National Park^74^ was higher in the early dry season compared to the early wet season. Over the dry season, lemurs experience conditions of relatively low temperatures and food and water restriction, especially in the dry western parts of Madagascar. This nutritional stress may result in a narrower diet, and the microbiota would be more precisely adapted to the food items available. This more restricted diet during the dry season could, therefore, explain the gradual decrease in microbiota richness that we observed. Such dietary change might lead to an altered microbial composition, which potentially facilitates the digestion of specific food items. It is tempting to speculate that this could also lead to an increased caloric intake, which might contribute to increased fitness of both the host and microbiota^21^.

The presence of different fruit trees results in large dietary differences across populations^75, 76^. For example, the four most predominant food items consumed by *E. fulvus* in Ankarafantsika in the early and early wet season, were *Buddleja madagascariensis, Psychotria* sp., *Vitex perrieri* and *Diospyros tropophylla*)^75, 77^, species that do not occur in Nosy Tanikely or Andasibe^76^. Furthermore, introduced mango trees (*Mangifera indica*) are only consumed at Nosy Tanikely. However, there is also some dietary overlap across populations, i.e., *Dichapetalum leucosia* and *Landolphia myrtifolia* were consumed by *E. fulvus* in both Ankarafantsika and Andasibe. Despite the overlap in some fruit species, the geographically separated populations of this lemur species showed major dietary differences, probably leading to major variations in microbiota composition in these populations.

We found a slight, but not significant, indication that parasite infections in the dry regions of Madagascar showed larger seasonal contrasts compared to the eastern rainforest. Another study found a higher parasite richness in areas with a large precipitation range throughout the year^78^. Many parasites require a certain temperature and humidity to complete their life cycles^78^ or as microhabitats for their larva^79^. The drier conditions towards the end of the dry season can prevent egg development and can lead to desiccation of the fragile eggs^31^. However, some related nematode species are able to survive such short periods of drought by entering a state of hypobiosis, until humidity conditions improve to the point where free-living larval stages can survive^80^. In addition to these direct seasonal influences on parasites, the lemur host influences these infection patterns as well. The host’s resource use and diet, in general, are considered as major determinants of host exposure to parasites^31^. It was also experimentally established that host foraging ecology has important consequences for the exposure to and transmission of parasites^81^. Food scarcity for lemurs is relatively high towards the end of the dry season^82, 83^ and the associated nutritional stress can have a repressive effect on the hosts’ immune system, which may result in a higher susceptibility to parasite infection^40^.

Seasonal changes in lemur reproductive status can also lead to changes in parasite infections patterns^84^. The early dry season coincides with the mating season of *Eulemurs*^85^, and more frequent physical contact both within and between lemur groups during this period may enhance parasite infection^84^. Besides, androgen and glucocorticoid levels of the males and oestrogen levels of the females increase during the mating season, which can lead to a higher susceptibility to parasite infections due to their repressive effect on the immune system^86^. Furthermore, the early wet season coincides with the weaning season, a season that is energy demanding, especially for lactating females. These behavioural and physiological differences may thus lead to differences in parasite infection status across different seasons. This hypothesis has yet to be explored with data to consider the likelihood of particular explanatory factors.

We also did not find an interactive effect of the two nematode species as coinfection appears to be independent. *Lemuricola* and *Callistoura* spp. colonise distinct parts of the gastrointestinal tract of their hosts, the caecum-colon and ileum-colon respectively^68^, which can explain the lack of interactions between these two species.

We hypothesised the microbiota composition to be altered and parasite infection prevalence to be increased in lemurs whose habitat is restricted to more intensely logged forests. For the microbial composition, we found statistically significant variation between samples taken at a previously logged and at a less disturbed site. Moreover, a higher richness of microbial consortia was observed in the logged area. Other studies that have addressed the impact of anthropogenic disturbance on the gut microbiota of wild primates seem to contradict our findings. For example, habitat disturbance was reported to lead to reductions in *Alouatta* gut microbial diversity^22^, and a similar pattern was found in Udzungwa red colobus monkeys^14^. These results may reflect a general trend of habitat degradation and reduced diversity in the ecological pool of microbial taxa available to colonize hosts^22^. However, the number of studies in this field is minimal. Besides, the type and intensity of anthropogenic disturbance and the forests’ regeneration time may be important as well^87^. Logging in our sites occurred nearly thirty years ago, and sites have been regenerating since^88^, which can explain the deviating patterns that were found in this study. Nevertheless, these forests still differ to a large extent in their structural characteristics, as well as tree species composition^89^, which may explain the differences in microbiota composition we found.

We found a relatively high abundance of Cyanobacteria in the *Eulemur* population in the less disturbed compared to the previously logged site. Sequences identified as Cyanobacteria are most probably derived from their non-photosynthetic gut dwelling siblings^90^. Even though they are part of the normal gut microbiota of mammals, it is not clear which role they play in intestinal ecosystems.

Concerning parasites, the prevalence of *Lemuricola* spp. was significantly higher in the more intensely disturbed site compared to the less disturbed site, while *Callistoura* spp. prevalence showed no such pattern. Selective logging results in a suite of alterations that may increase infection risk and susceptibility to certain parasite infections in resident populations^39^. For example, studies on howler monkeys have reported higher GI parasite diversity and abundance in primates inhabiting degraded areas compared to those in less disturbed areas^91^. The depletion of the GI microbiota in degraded environments may explain these patterns. However, other studies show only minimal effects of disturbance on patterns of intestinal parasite infection^92^. As mentioned above, our logged forest site has been regenerating over decades, and it seems that lemurs have been able to adapt to differences in food availability and forest structural differences accordingly^89^. As eggs of *Lemuricola* spp. are deposited in the perianal region of their host^68^, body contact and grooming behaviour may be important factors in explaining the prevalence of this nematode within a population. Interaction rates and local lemur densities may be increased, and home ranges more restricted in the more intensely logged forest, which has been shown to increase parasite infection risks^40, 93^. This may explain the higher *Lemuricola* spp. prevalence we found in these forests.

Several other studies observed a relationship between microbiota and GI parasites^21, 48–51, 54^. We found a small but significant correlation of microbial composition with the prevalence of *Callistoura* spp. In addition, the lemur population in Ankarafantsika had a significantly lower infection prevalence of *Callistoura* spp. compared to lemur populations in other areas and at the same time, this population showed the highest microbiota richness. Despite the statistical significance of the correlations, interpretation of this correlations should be made with care. On the one hand, GI parasites can have a direct influence on intestinal microbiota by damaging the host’s intestinal epithelium, extracting nutrients in the GI tract^59^, secreting antimicrobial products or inducing an inflammatory response^94^. On the other hand, observed correlations could not provide direct evidence for these mechanistic aspects. The microbiota is a dynamic ecosystem that has been shown to be affected by a broad range of environmental factors. However, the effect of factors with smaller relative weight is often masked by individual-specific factors like diet and genetic background^95^. This could incorrectly reflect the true importance of such minor factors, particularly in wildlife studies where individual variation cannot easily be controlled.

Several studies found that the presence of some nematode species is linked to high microbiota diversity, with potential beneficial consequences for host health^47, 58, 60, 94, 96^. It is assumed that the GI microbiota regulate the immune system, but also that GI nematodes can alter the bacterial composition and structure, thereby creating conditions that can facilitate nematode infestations^96^. Although it has been shown that some parasites change environmental conditions prevailing in the intestine, and thus affecting also microbial habitats, the exact relations between parasites and the microbiota remain unclear^97^. Most parasite species, and directly transmitted parasites in particular, co-evolve in association with only a few host species and adapt to the host gut environment and diet, resulting in host-driven diversification^98^ that allowed to speculate about microbe-parasites evolutionary crosstalk. Understanding underlying mechanisms is critical for improving our knowledge of parasite–microbe interactions in wild primate populations. This can become achievable with a greater longitudinal sampling effort, refined, standardised sample preservation protocols, genetic identification of the nematodes with molecular methods, and if possible in vitro and in vivo model experiments.

In conclusion, this study investigated the impact of seasonality and past logging on host-associated parasite infections, faecal bacterial communities, and correlative patterns between these GI inhabitants in geographically separated *Eulemur* populations. Our results show that seasonal differences and past logging events significantly contributed to explaining the observed temporal variations in parasite infections and microbial diversity. The variation in microbiota composition at the genus level showed a significant correlation with the presence of parasites, suggesting a relationship between gastrointestinal parasites and microbiota composition under natural conditions. The factors that influence microbiota composition and presence of parasites may, in turn, affect host nutrition, behaviour, and health. These findings likely apply to other wild mammal communities as well. We believe it is crucial to consider the potential role of microbiome-parasite associations on the hosts’ GI stability, health, and survival.

## Methods

### Study site

Our research was performed in eight geographically distinct sites (Fig. 1, Table 1). Kirindy Forest, Ankarafantsika National Park (NP), and Zombitse NP are located on the western, north-western, and south-western side of Madagascar, respectively. They consist of dry deciduous forest with pronounced seasonality ^99^. These western regions have a higher annual mean temperature than the eastern rainforests but receive less rainfall.

**Figure 7:**
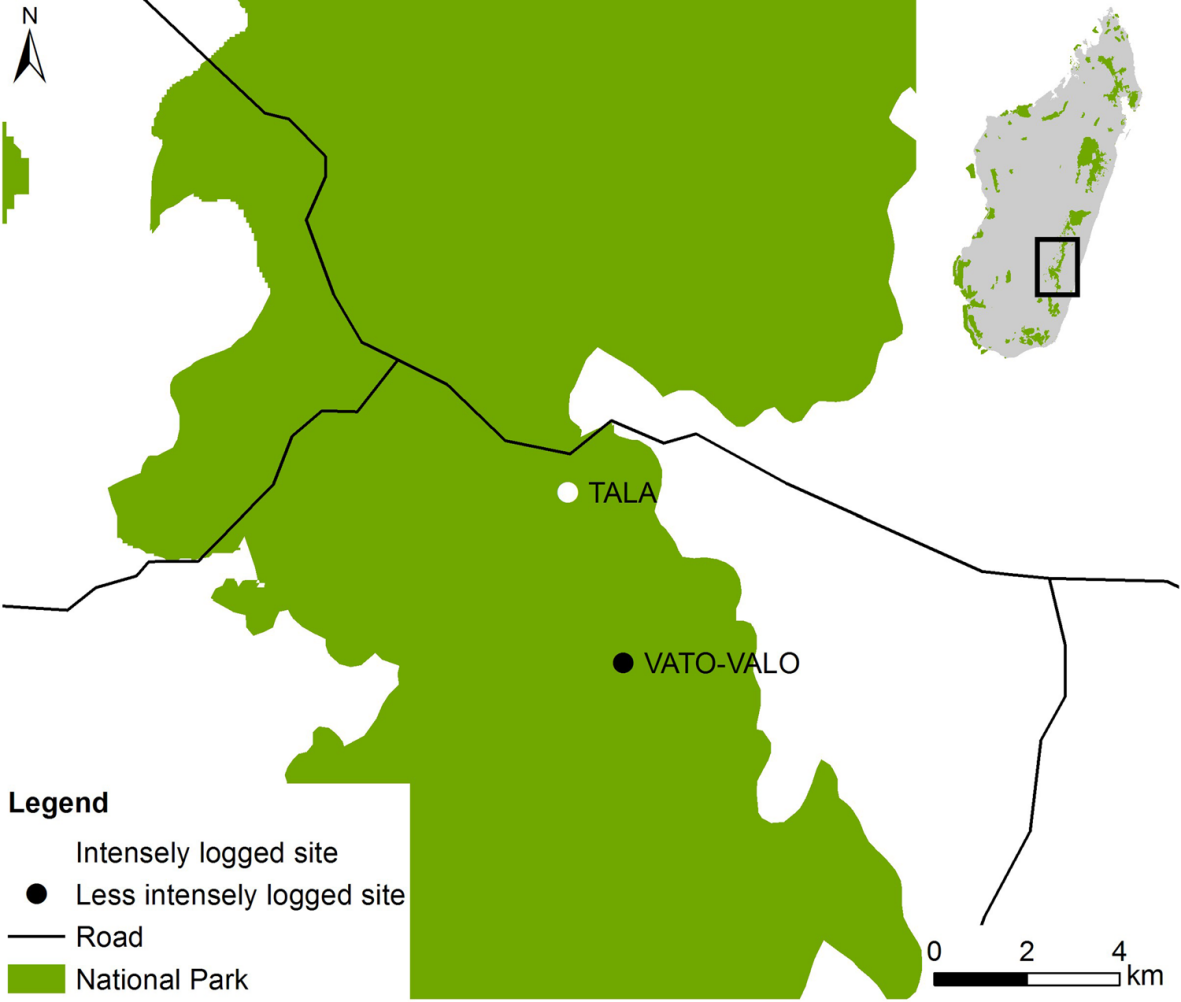
Map of Ranomafana National Park and the two forest sites that were surveyed in this study. Talatakely (white dot) experienced relatively intense logging in the past, while Vatoharanana- Valohoaka (black dot) experienced no such disturbances. This map was generated via ArcGIS version 10.5. Data was downloaded from UNEP-WCMC and IUCN (2016), Protected Planet: [National Parks of Madagascar; The World Database on Protected Areas (WDPA) [On-line], [May 2016], Cambridge, UK: UNEP-WCMC and IUCN.

In contrast, Andasibe Mantadia NP and Ranomafana NP are located on the eastern side of Madagascar. They are relatively wet rain forests with a less distinct dry season compared to the western areas^100^. Within Ranomafana NP, we distinguished two research sites, Talatakely (TALA) and Vatoharanana-Valohoaka (VATO-VALO) with different degrees of anthropogenic disturbance (Fig. 7)^89, 101^. Before the establishment of the national park in 1991, the forests in this area were used by local inhabitants, amongst others for slash-and-burn agriculture^100^. Now, more than 25 years after the last logging activities, Ranomafana NP shows a high heterogeneity in forest structure.

The islands Nosy Be, Nosy Komba, and Nosy Tanikely, are located in the north-west of Madagascar. The forests of Nosy Be (~ 320 km^2^) are largely replaced by coffee, fruit, and ylang-ylang plantations, and by rice and sugar cane fields. Only Lokobe NP (~ 7 km^2^) at the south-eastern part of the island still contains the island’s original forest vegetation. Nosy Komba and Nosy Tanikely are located in between Nosy Be and the mainland. The vegetation on Nosy Komba (~ 25 km^2^) is similar to Nosy Be. The vegetation at Nosy Tanikely (~ 0.06 km^2^) mainly consists of low forest and bushy vegetation, including palm trees and planted banana and mango trees, surrounded by a sandy shore with large rock formations (de Winter, *pers. obs.*^102^).

### Study species

True lemurs (genus *Eulemur*, family Lemuridae) are medium-sized (body and tail length 30–50 cm, 2–4 kg) arboreal primates that occasionally move on four legs on the ground. They live in social groups ranging from two to fifteen individuals, and their diet primarily consists of fruits, flowers, and leaves^62^. We studied four *Eulemur* species: *Eulemur rufifrons, E. fulvus, E. macaco*, and *E. rubriventer*. The main difference between the *Eulemur* species is their group size *E. rufifrons, E. macaco,* and *E. fulvus* live in multi-male, multi-female groups from four to 18 individuals^103–105^, whereas E. rubriventer lives in small monogamous groups from two up to five individuals^106^. *Eulemur rufifrons* lives in the southwest and east, and the native range of *Eulemur fulvus* is in the north of Madagascar, on both the east and west side^107^. This species has also been introduced to the northern island Nosy Tanikely. *Eulemur macaco* is found on the mainland and several islands in the north-west, while *Eulemur rubriventer* inhabits forests in eastern Madagascar (Fig. 1, Table 1). *Eulemur rubriventer* and *E. rufifrons* live sympatrically in Ranomafana NP^108^.

### Faecal sample collection

We collected 338 faecal samples between October 2013 and February 2015 (Table 1), of which 133 were also used in a previous study^64^. Within Ranomafana NP, we collected 103 samples; 38 samples from a previously logged site (Talatakely) and 65 from a site that was considered a less disturbed site in terms of its logging history (Vatoharanana-Valohoaka). Immediately after defecation, fresh faecal samples (3–4 g) were collected non-invasively. We noted visual characteristics, i.e., consistency, colour, presence of blood, mucus, or tapeworm proglottids. We also reported GPS coordinates, time, group size, group composition, age (sub-adult if ≤ 2 years old or adult if ≥ 3 years old), and sex. We allocated a body fur condition score to the individuals whose faeces were collected^109^. We aimed at sampling all adults within a social group, and we did not resample the same individuals. When encountering a new social group, we first recorded as many distinguishable features as possible of all lemur individuals. As soon as we were not sure whether the faeces were from a new individual or whether we already sampled the animal, we moved on to another group. As we worked mostly within National Parks or Reserves, the lemurs were all habituated to human observers, mainly due to the frequent visits by tourists or researchers, which facilitated the faecal collection. We found no abnormalities in the consistency and colour of the faeces and we did not find blood, mucus or tapeworm proglottids in any of the faecal samples. Within 12 h after collection, each faecal sample was divided over two sterile tubes: one gram of faeces was stored in a tube filled with 5 ml of 70% ethanol (Supplementary Material S2) and two grams of faeces was placed in a tube filled with 15 ml SAF fixative^44, 110^. Samples were analysed at the Laboratory of Microbiology, Wageningen University & Research, and the Department of Infectious Diseases and Immunology, Utrecht University. All described methods were performed in accordance with the relevant guidelines and regulations and were approved by the trilateral commission (CAFF/CORE) in Madagascar (permits 297/13 and 143/14/MEF/SG/DGF/DCB.SAP/SCBSE).

### DNA-based bacterial composition analyses

Faecal bacterial microbiota composition, determined by next-generation sequencing of 16S rRNA gene fragments, was used as a proxy for the intestinal microbial community. We extracted microbial DNA from the faecal samples collected in Ranomafana NP following a modified double bead-beating procedure using the QIAamp® DNA Stool Mini Kit (Qiagen, Valencia, CA, USA) (Based on^111^). For the sample processing, we used the protocol proposed by Yu and Morrison (2004), modified by^112^. Prior to DNA extraction, faecal material was air-dried during 15–20 min in a fume hood to remove ethanol from samples. We extracted DNA from samples collected at the other sites using the Maxwell® 16 Research Instrument (Promega, Madison, USA) in combination with the corresponding RNA extraction kit customised for faecal DNA extraction according to manufacturer’s instructions. Prior to DNA extraction, samples were rehydrated through a series of ethanol solutions with decreasing proportions of ethanol in steps of 10%. For rehydration, 1.5 ml of 70% ethanol with faecal particles was transferred into a fresh 2 ml tube and centrifuged at 13,000 rpm for 5 min. After centrifugation, part of the supernatant was replaced with the same amount of distilled water to decrease ethanol concentration by 10%, vortexed, and incubated for 10 min at RT. These steps were repeated until the ethanol was entirely replaced by distilled water. Cell disruption and lysis were performed as described above, but instead of lysis buffer, we used S.T.A.R buffer (Roche Molecular Systems, USA). DNA quality and concentration were spectrophotometrically verified (Nanodrop Technologies, Wilmington, USA. For each sample, barcoded amplicons were generated from 40 ng of extracted DNA using a two-step PCR method in a LabCycler Gradient (SensoQuest, Germany) and pooled afterwards as described previously^113^. Briefly, the V1–V2 region of the 16S rRNA was first amplified by PCR (25 cycles of 95 °C (30 s), 52 °C (40 s), and 72 °C (90 s)), followed by post-elongation (72 °C, 7 min) using primer pair 27F–DegS: 5′-GTTYGATYMTGGCTCAG-3′^114^ and 338R–I: 5′-GCWGCCTCCCGTAGGAGT-3′/338R–II: 5′-GCWGCCACCCGTAGGTGT-3′^115^ that contained forward and reverse linkers UniTag I (5′-GAGCCGTAGCCAGTCTGC-3′) and UniTag2 (5′-GCCGTGACCGTGACATCG-3′), respectively. Amplicons were then used as a template for a second PCR in order to introduce sample-specific barcodes, using individual barcode primers targeting Unitag1 and UniTag2 sequences. The amount and size of the amplicons were checked visually by agarose gel electrophoresis. The PCR products were purified using the HighPrepTM PCR kit (MagBio Genomics), concentrated using magnetic beads (MagBio, Switzerland) according to the HighPrep protocol, quantified using the Qubit dsDNA BR Assay Kit (Life Technologies, USA), and pooled in equimolar amounts into libraries of 48 samples, including two mock communities of defined composition, for paired-end sequencing (300 bp) on the Illumina Miseq platform at GATC Biotech (Konstanz, Germany; now part of Eurofins Genomics Germany GmbH). Mock communities i.e. mixes of quantified and purified copies of bacterial 16 s rRNA genes in known proportions, are routinely used in our laboratory to assess quality and reliability of a sequencing run, amplicon preparations, and quality of data processing, as was described previously^116^.

The amplicon sequences were demultiplexed, and the subsequent analysis of raw rRNA gene sequence data was performed using NG-Tax^116^. Reads assigned to OTUs of plant origin such as chloroplast and plant mitochondrial DNA were removed from the dataset used for downstream analysis. The raw data was ranked per individual sample based on the matching of reads to OTUs, allowing an error of one nucleotide.

### Parasite isolation

The collected faecal samples were examined for the presence of GI nematodes with the use of the Centrifugation-Sedimentation-Flotation (CSF) method^117^. GI nematode species identification was based on morphological traits such as colour, shape, size, and content of eggs^68, 84, 118^. A rough estimation of the number of parasite eggs per gram of faeces (EGP) was obtained by simple counts. Since the number of eggs that end up in the faeces is not a reliable index of adult worm burden^119^, the egg count cannot be regarded as a measurement of infection intensity, but rather as a measurement of infectivity.

### Statistical analysis

After initial sequence data processing with NG-tax, we combined the OTU table, metadata, and phylogenetic tree into a “phyloseq” object, as implemented in the “phyloseq” R package (v.1.22.3). Further analyses were carried out in R (v 3.4.1) (Supplementary Material S2). OTUs that were encountered in less than three samples, OTUs not assigned to any taxonomic level (NA) and OTUs identified as chloroplast and mitochondria were removed. In addition, samples with a low number of the reads (less than 1000 reads), missing metadata of interest, and one sample (i.e., ‘NT9F’, due to the low quality of the starting material) were removed from the data set (Supplementary Material S1, Table S2, and Supplementary Material S2). For beta diversity analysis, the weighted UniFrac distance matrix was calculated from the OTU table and phylogenetic tree as implemented in the “phyloseq” package, with the phylogenetic tree rooted at the midpoint (package “phangorn”). Multidimensional scaling with weighted UniFrac as a distance matrix (PCoA) was applied (package “phyloseq”) to obtain a first insight into the beta diversity of faecal microbial communities in the investigated lemur populations. We used dbRDA (dist.wu ~ Site + Species + Fieldseason + CallPrev + Sex + Age + LemPrev) and an ANOVA like permutation test (anova.cca; permutations = 9999) to identify variables that significantly contribute to explaining the observed variation in microbial composition (package “vegan”). To this end, we employed dbRDA to enhance visualization of the results when the focus shifted from providing an overall picture to investigating individual factors. Constrained ordination techniques, such as dbRDA, allow researchers and readers to zoom in on a two-dimensional representation with the best separation of samples. Variable “Social Group” was excluded from the analysis due to extremely uneven sample distribution (Supplementary Material S2, Tables S1, S3), with 28 out of a total of 92 social groups including only one sample. The degree to which individual factors could explain microbiota composition was estimated by partial dbRDA with control for variables that were not used as a constraint. R^2^ values were used as an estimator of variation explained by a constraint (package “vegan”). Phylogenetic diversity was used as a primary alpha diversity measure. It was calculated from the phyloseq object with the OTU table rarefied at a read depth of 1051, using a custom function (author Thomas W. Battaglia, https://github.com/twbattaglia). Statistical differences between alpha diversity of pre-defined sample groups were assessed by posthoc Kruskal Nemenyi-tests (package “PMCMR”). The datasets generated during this study are available in the Supplementary Material S1 and the public read archive EBI, study name ‘ena-STUDY-WAGENINGEN UNIVERSIT-03-04-2017–14:57’, with accession number ‘PRJEB20227’ (link: https://www.ebi.ac.uk/ena/data/view/PRJEB20227).

To analyse the effect of seasonality (early dry vs early wet) and location (western dry deciduous forests vs eastern rainforests) on the infection prevalence of *Callistoura* and *Lemuricola* spp. in *Eulemur* species, GLMMs were used, assuming a Binomial distribution and logit link function for data aggregated per social group. We included random effects for sites within a location and observation-level random effects for social groups, and fixed effects for species, season, location, and the interaction between season and location. The observation-level random effects handle possible binomial overdispersion. The factor species entered the model as a control variable to avoid confounding of location effects with species effects. We focused specifically on the interaction between location and season to test the seasonality hypothesis as formulated in the Introduction. To present estimated infection prevalence with 95% confidence intervals (CI) on the probability scale, we back-transformed the results (on the logit-scale) from the GLMMs first. Next, we applied a shrinkage factor^120^, which is needed for GLMMs, to obtain predicted population means instead of medians. To test whether infections by the two nematode genera occurred independently, we modified the GLMM for *Callistoura* spp., using unaggregated data, by adding an indicator variable for *Lemuricola* spp. as a regressor to the model. In this way, we allowed the infection prevalence for *Callistoura* spp. could be different among lemurs with or without *Lemuricola* spp. infections.

In a subset of the data (Ranomafana NP; N = 103 individuals of *E. rubriventer* and *E. rufifrons* only), we analysed *Callistoura* and *Lemuricola* spp. infection prevalence comparing disturbed and less disturbed subsites. Again, we aggregated infection scores per social group and used ordinary Generalised Linear Models (GLMs) assuming a binomial distribution for the number of infected animals per social group and logit link function. We entered effects for the control factor species and for the main factors of interest: disturbance (less vs more disturbed subsites), season (early dry vs early wet), and their interaction into the model. In the analysis of *Callistoura* spp. prevalence, a smaller model was fitted due to the low numbers of cases (14 cases, with just 1 in the less disturbed site). Extra-binomial variation could not be ruled out, because individuals within social groups may have correlated responses. Because of different group sizes (range 1–7), we used Williams’ method as available in the dispmod package of R^121^. If the overdispersion was not present, when judging the residual deviance, we used an ordinary binomial GLM. We calculated back-transformed predicted means presented with 95% CI for the previously disturbed and less disturbed sites.

Model assumptions were checked by inspection of residuals, leverages, and collinearity statistics, and model stability (for GLMM) and dfbetas (for GLM) were assessed. After model checking, comparisons of the full model (separately for the analyses of *Callistoura* and *Lemuricola* spp. infections, seasonality, and disturbance analyses) with reduced models were made using likelihood ratio tests (LRT), followed by tests for individual factors in case of significant results. Regardless of the results from omnibus tests, we tested the specifically formulated hypotheses regarding seasonality and forest disturbance (see “Introduction”). Pseudo R^2^^122^ for the full models were calculated (results are shown in Supplementary Material S1, Table S1).

We performed the statistical analyses in base R^123^, using the R packages lme4 for the GLMMs^124^ and emmeans^125^ for prediction of group means (Supplementary Material S2), with car^126^ for variance inflation factors, DHARMAa^127^ for residual checking in GLMMs, and MuMIn^128^ for creating pseudo R^2^ values (elaborate results of statistical analyses are presented in the Supplementary Material S2).

## Supplementary information

Supplementary Material S1.

Supplementary Material S2.
