# Supplementary material for: Effects of seasonality and previous logging on faecal helminth-microbiota associations in wild lemurs
Source: Sci Rep. 2020 Oct 8;10:16818. doi: 10.1038/s41598-020-73827-1 (PMC7544911; doi:10.1038/s41598-020-73827-1)
Supplement: Supplementary file 1 — Supplementary Material S1. [file 41598_2020_73827_MOESM1_ESM.pdf]

Effects of seasonality and previous logging on faecal helminth-microbiota associations in wild lemurs

de Winter, I.I. [1*/**], Umanets, A. [2*], Gort, G.[3], Nieuwland, W.[4], van Hooft, P.[4], Heitkönig, I.M.A.[4], Kappeler, P.M.[5], Prins, H.H.T.[4], Smidt, H.[2]

Affiliations

[1] Utrecht University, Padualaan 8, 3584 CH Utrecht | The Netherlands

[2] Laboratory of Microbiology, Wageningen University & Research, Stippeneng 4, 6708 WE Wageningen, The Netherlands

[3] Biometris, Wageningen University Plant Research, Droevendaalsesteeg 1, 6708 PB Wageningen, The Netherlands

[4] Resource Ecology Group, Wageningen University & Research, Droevendaalsesteeg 3a, 6708 PB Wageningen, The Netherlands

[5] Behavioral Ecology and Sociobiology Unit, German Primate Center, Kellnerweg 4, 37077 Göttingen, Germany

[*] Authors contributed equally

[**] Corresponding author. Correspondence to: i.i.dewinter@uu.nl

Callistoura and Lemuricola prevalence in Eulemurs: seasonal contrast between West and East Madagascar

Gerrit Gort and Iris de Winter

16-07-2018

Read parasites data

```
parasites <- read_excel("2017_11_20_Supplementary Dataset S1.xls",
                        sheet="Supplementary Dataset S1", na="U")
Qarea <- data.frame(parasites[,
          c("Site","SpecificLoc","Species","Group","Season","CalPrev","LemPrev")])

Qarea$Loc <- factor(Qarea$Site %in%
  c("Ankarafantsika","Kirindy","Zombitse","Nosy_Be","Nosy_Komba","Nosy_Tanikely"))
levels(Qarea$Loc) <- c("East", "West")

Qarea$Season <- factor(Qarea$Season)
levels(Qarea$Season) <- c("Dry", "Wet")

Qarea$Site   <- factor(Qarea$Site)
Qarea$Group  <- factor(Qarea$Group)

Qarea$CalLemPrev <- factor(2*(Qarea$CalPrev)+Qarea$LemPrev)
levels(Qarea$CalLemPrev) <- c("Neither", "LemOnly", "CalOnly", "Both")
Qarea$CalLemBothPrev <- as.numeric(Qarea$CalPrev==1 &  Qarea$LemPrev==1)

Qarea <- droplevels(Qarea)
head(Qarea)
```

```
##         Site SpecificLoc     Species Group Season CalPrev LemPrev  Loc
## 1 Ranomafana        Tala Rubriventer     1    Wet       1       1 East
## 2 Ranomafana        Tala Rubriventer     1    Wet       1       0 East
## 3 Ranomafana        Tala Rubriventer     5    Wet       0       0 East
## 4 Ranomafana        Tala Rubriventer     3    Wet       0       0 East
## 5 Ranomafana        Tala Rubriventer     6    Wet       1       0 East
## 6 Ranomafana        Tala Rubriventer     5    Wet       0       1 East
##   CalLemPrev CalLemBothPrev
## 1       Both              1
## 2    CalOnly              0
## 3    Neither              0
## 4    Neither              0
## 5    CalOnly              0
## 6    LemOnly              0
```

Aggregate data to level of social group

```
Qarea.grp <- summaryBy(CalPrev + LemPrev + CalLemBothPrev ~
            Season + Loc + Site + Species + Group, data=Qarea, FUN=c(sum, length))
Qarea.grp <- Qarea.grp[,-c(10,11)]
names(Qarea.grp)[6:9] <- c("k.c","k.l","k.cl","n")
head(Qarea.grp)
```

```
##   Season  Loc       Site     Species Group k.c k.l k.cl n
## 1    Dry East   Andasibe      Fulvus    13   0   0    0 1
## 2    Dry East   Andasibe      Fulvus    14   2   0    0 5
## 3    Dry East   Andasibe      Fulvus    15   3   1    0 5
## 4    Dry East   Andasibe      Fulvus    16   1   0    0 1
## 5    Dry East   Andasibe      Fulvus    17   1   0    0 2
## 6    Dry East Ranomafana Rubriventer    44   2   1    1 2
```

Descriptive statistics: frequency tables

```
# summary statistics for group sizes
length(Qarea.grp$n)
```

```
## [1] 95
```

```
summary(Qarea.grp$n)
```

```
##    Min. 1st Qu.  Median    Mean 3rd Qu.    Max.
##   1.000   1.000   2.000   3.526   5.000  15.000
```

```
# group sizes range 1-15 with average 3.5

# frequency table for location by season
addmargins(xtabs(n ~ Loc + Season, data=Qarea.grp))
```

```
##       Season
## Loc     Dry Wet Sum
##   East   60  99 159
##   West   69 107 176
##   Sum   129 206 335
```

```
# frequency table for site by species
addmargins(xtabs(n ~ Site + Species, data=Qarea.grp))
```

```
##                 Species
## Site              Fulvus Macaco Rubriventer Rufifrons Sum
##   Andasibe            43      0           0         0  43
##   Ankarafantsika      50      0           0         0  50
##   Kirindy              0      0           0        37  37
##   Nosy_Be              0     18           0         0  18
##   Nosy_Komba           0     23           0         0  23
##   Nosy_Tanikely       17      0           0         0  17
##   Ranomafana           0      0          68        48 116
```

```
##   Zombitse             0      0             0        31  31
##   Sum                110     41            68       116 335
```

```r
# notice the unbalancedness: in 7 out of 8 sites only a single species is observed

ftable(xtabs(n ~ Species + Loc + Season, data=Qarea.grp))
```

```
##                     Season Dry Wet
## Species     Loc
## Fulvus      East            14  29
##             West            32  35
## Macaco      East             0   0
##             West             0  41
## Rubriventer East            21  47
##             West             0   0
## Rufifrons   East            25  23
##             West            37  31
```

```r
# notice that Macaco is only observed in Wet season (in Western region)

# frequency tables of parasites prevalences for Callistoura, Lemuricola and both
addmargins(xtabs( ~ Site + CalPrev, data=Qarea))
```

```
##                   CalPrev
## Site                 0   1 Sum
##   Andasibe          17  26  43
##   Ankarafantsika    38  12  50
##   Kirindy            4  33  37
##   Nosy_Be            6  12  18
##   Nosy_Komba         6  17  23
##   Nosy_Tanikely      6  11  17
##   Ranomafana        33  83 116
##   Zombitse           3  28  31
##   Sum              113 222 335
```

```r
addmargins(xtabs( ~ Site + LemPrev, data=Qarea))
```

```
##                   LemPrev
## Site                 0   1 Sum
##   Andasibe          40   3  43
##   Ankarafantsika    45   5  50
##   Kirindy           29   8  37
##   Nosy_Be           15   3  18
##   Nosy_Komba        14   9  23
##   Nosy_Tanikely     14   3  17
##   Ranomafana       101  15 116
##   Zombitse          26   5  31
##   Sum              284  51 335
```

```r
addmargins(xtabs( ~ Site + CalLemPrev, data=Qarea))
```

```
##                   CalLemPrev
## Site               Neither LemOnly CalOnly Both Sum
```

```
##   Andasibe              16       1       24     2  43
##   Ankarafantsika        35       3       10     2  50
##   Kirindy                2       2       27     6  37
##   Nosy_Be                4       2       11     1  18
##   Nosy_Komba             3       3       11     6  23
##   Nosy_Tanikely          5       1        9     2  17
##   Ranomafana            29       4       72    11 116
##   Zombitse               2       1       24     4  31
##   Sum                   96      17      188    34 335
```

```
# frequency tables of parasite occurrences vs west vs season
ftable(xtabs( ~ Loc + CalPrev + Season, data=Qarea))
```

```
##               Season Dry Wet
## Loc  CalPrev                
## East 0                18  32
##      1                42  67
## West 0                28  35
##      1                41  72
```

```
ftable(xtabs( ~ Loc + LemPrev + Season, data=Qarea))
```

```
##               Season Dry Wet
## Loc  LemPrev                
## East 0                52  89
##      1                 8  10
## West 0                55  88
##      1                14  19
```

```
ftable(xtabs( ~ Loc + CalLemPrev + Season, data=Qarea))
```

```
##                  Season Dry Wet
## Loc  CalLemPrev                
## East Neither             16  29
##      LemOnly              2   3
##      CalOnly             36  60
##      Both                 6   7
## West Neither             22  29
##      LemOnly              6   6
##      CalOnly             33  59
##      Both                 8  13
```

Main summary statistics:

- 95 social groups, with group size range 1 - 15; average group size = 3.5, median size = 2.
- 335 individual lemurs, approximately evenly split over East and West, but slightly more in wet season compared to dry season.
- 4 Eulemur species: E. Fulvus, E. Macaco, E. Rubriventer and E. Rufifrons.
- Species distribution very uneven over sites, as some species only occur in some sites.
- E. Macaco only observed in wet season (in West).
- Callistoura prevalence: 222 out of 335 (66%), occurring in all sites; Lemuricola prevalence: 51 out of

335 (15%), occurring in all sites; joint prevalence of Callistoura and Lemuricola: 34 out of 335 (10%).

Analysis of Callistoura infection prevalence

We apply a GLMM for (aggregated) binomial data with fixed effects location (East - West), season (early dry - early wet), location by season interaction, and control variable species. Random effects are added for Sites and at observation-levelto account for variation between social groups within sites (extra-binomial variation), see e.g. Harrison XA (2014): Using observation-level random effects to model overdispersion in count data in ecology and evolution.

Callistoura: assumption checking for GLMM

The full model, described above, is fitted, for which we check assumptions.

```
C.FM <- glmer(cbind(k.c, n-k.c) ~
                Species + Loc + Season + Loc:Season + (1 | Site / Group),
              contrasts=list(Species=contr.sum, Loc=contr.sum, Season=contr.sum),
              family=binomial, data=Qarea.grp)

# Pearson residuals and linear predictor values for residual plots
par(mfrow=c(1,2))
res <- residuals(C.FM,  type="pearson")
lp  <- predict(C.FM)
qqnorm(res) # just to check for outliers, no normality assumed
plot(res ~ lp, data=Qarea.grp)
```

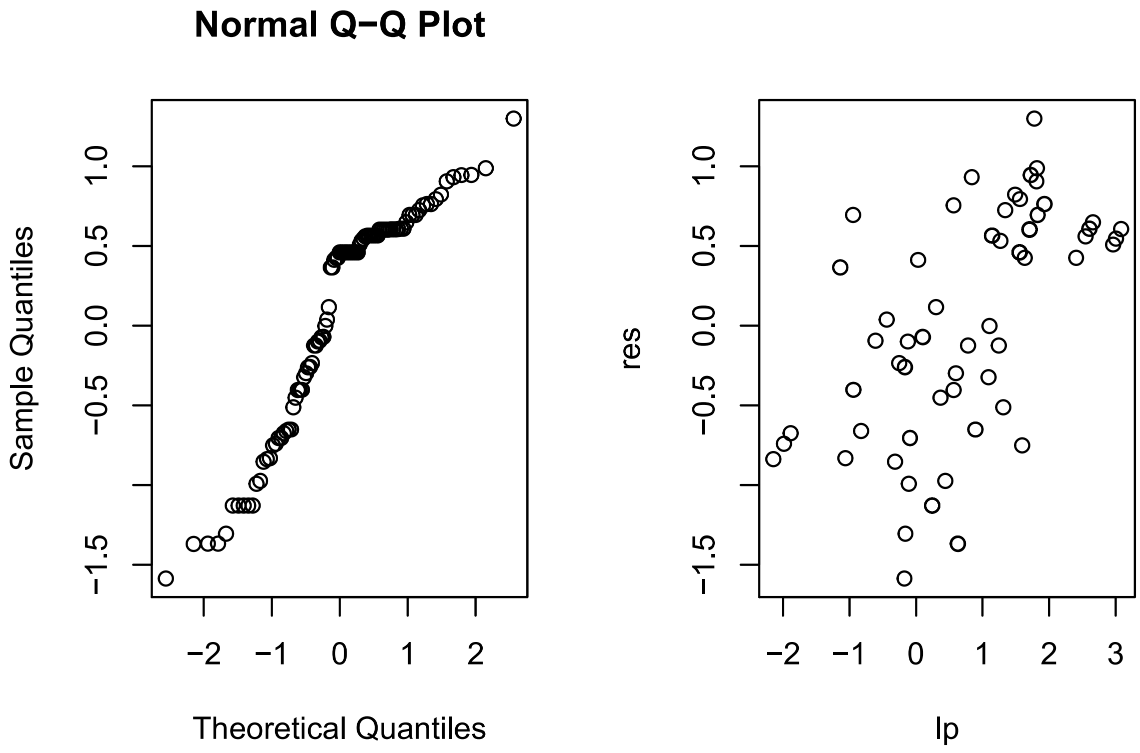


```
par(mfrow=c(1,1))
# Neither outliers are found, nor extreme patterns in residual plot.
# Typical systematic trends can be observed, as expected in residual plots for
# random effects models (e.g. at fixed value of linear predictor the average value
# of residuals is not zero).

# Another model check based upon residuals in GLMMs uses DHARMa, see Hartig
# (2018) DHARMa: Residual Diagnostics for Hierarchical (Multi-Level / Mixed)
# Regression models. R package version 0.2.0.
set.seed(123)
simulationOutput <- simulateResiduals(fittedModel = C.FM, n=5000)
testUniformity(simulationOutput=simulationOutput)
```

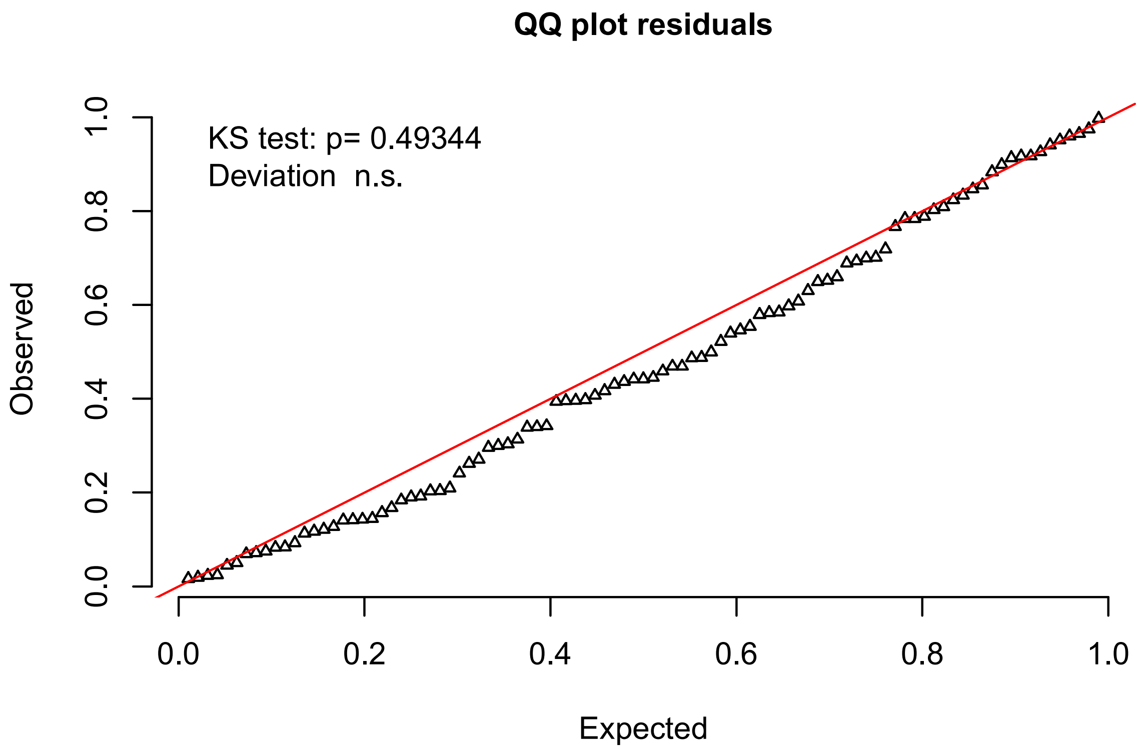


```
##
##  One-sample Kolmogorov-Smirnov test
##
## data:  simulationOutput$scaledResiduals
## D = 0.085337, p-value = 0.4934
## alternative hypothesis: two-sided
```

```
# Residual plot looks good, no indications of problems; test statistic
# (Kolomogorov-Smirnov) indicates no problem.

# To get a rough idea about leverage, we calculate leverages as would be used in
# GLM. We don't know of a method for GLMM's. Instead of GLMM fit an ordinary GLM:
C.lev <- hatvalues(glm(cbind(k.c, n-k.c) ~ Species + Season + Loc + Season:Loc,
                    family=binomial, data=Qarea.grp))
range(C.lev)
```

```
## [1] 0.01722076 0.25944469
```

```
(cutoff.lev <- 2*7/nrow(Qarea.grp)) # threshold for leverage in linear models
```

```
## [1] 0.1473684
```

```
sum(C.lev > cutoff.lev)
```

```
## [1] 10
```

```
# Largest leverage is 0.26, larger than the threshold 0.15 (=2*(7/95)), as would
# be used in LM; 10 observations have leverages larger than threshold.
```

```r
Qarea.grp$n[C.lev > cutoff.lev]
```

```
##  [1]  5  5 15 11 10 10  9 10  9 12
```

```r
# All high leverage observations have large group sizes; they should indeed
# have relatively high leverage, because the binomial variance of these
# observations is relatively small. We are not worried about this result.

# To get a rough idea about collinearity among fixed factors, we calculate the
# generalized variance inflation factor, as would be used in LM using function
# vif from the car package. We don't know of a method for GLMM's.
vif(glm(cbind(k.c, n-k.c) ~ Species + Season + Loc + Season:Loc,
        family=binomial, data=Qarea.grp))
```

```
##                GVIF Df GVIF^(1/(2*Df))
## Species    1.974705  3        1.120083
## Season     2.237788  1        1.495924
## Loc        2.899314  1        1.702737
## Season:Loc 4.193295  1        2.047754
```

```r
# Largest GVIF2 = 2.05 for Season:Loc, which is relatively small.

# We check for model stability using a script kindly made available by
# dr. Roger Mundry from the Max Planck Institute of Evolutionary Anthropology.
source("D:/Data/Iris de Winter/parasites/Mundry/glmm_stability.r")

# Nesting of random factors (Group within Site) doesn't seem to be handled
# properly by script. We circumvent the problem by adding random effect of
# Group separately.

# We further define deviation regressors for fixed factors ourselves.
# Using contr.sum doesn't seem to work properly in the stability script.
Qarea.grp$SpFulvusD <-
  1*(Qarea.grp$Species=="Fulvus") -1*(Qarea.grp$Species=="Rufifrons")
Qarea.grp$SpMacacoD <-
  1*(Qarea.grp$Species=="Macaco") -1*(Qarea.grp$Species=="Rufifrons")
Qarea.grp$SpRubriventerD <-
  1*(Qarea.grp$Species=="Rubriventer") -1*(Qarea.grp$Species=="Rufifrons")
Qarea.grp$LocD <- 1*(Qarea.grp$Loc =="East") -1*(Qarea.grp$Loc =="West")
Qarea.grp$SeasonD <- 1*(Qarea.grp$Season =="Dry") -1*(Qarea.grp$Season =="Wet")
Qarea.grp$LSD <- Qarea.grp$LocD *Qarea.grp$SeasonD

C.FM2 <- glmer(cbind(k.c, n-k.c) ~ SpFulvusD + SpMacacoD + SpRubriventerD +
                 LocD + SeasonD + LSD  +  (1 | Site) + (1 | Group),
                 family=binomial, data=Qarea.grp)
full.stab <- glmm.model.stab(model.res=C.FM2, para=F, data=Qarea.grp)
```

```
## [1] "please carefully evaluate whether the result makes sense, and if not, please contact me"

## fixed-effect model matrix is rank deficient so dropping 1 column / coefficient
```

```r
round(full.stab$summary[, -1], 3)
```

```
##                          orig    min    max
## (Intercept)             1.147  0.567  1.396
## SpFulvusD              -1.341 -2.658 -0.097
## SpMacacoD              -0.814 -1.763 -0.228
## SpRubriventerD          1.320  0.923  2.264
## LocD                   -0.284 -1.154  0.519
## SeasonD                -0.240 -0.385 -0.103
## LSD                     0.238  0.008  0.391
## Group@(Intercept)@NA    1.059  0.668  1.295
## Site@(Intercept)@NA     0.773  0.000  0.956
```

```r
# We observe substantial model instability for Location (parameter estimates
# range from negative to positive values).
# Less instability is observed for some species (value zero is not included
# in their ranges).
# For main effect of season (averaged over species and locations) and
# interaction Location x Season (LSD) the model stability is highest.
# The interaction parameter is the parameter of main interest.
# Because we use the sum-to-zero parametrization, the main effect parameters
# for Location and Season represent half the difference (on logit scale)
# between the two groups, averaged over the levels of other factors.
# The interaction parameter represents a quarter of the usual interaction
# contrast, so 0.25((L1S1-L1S2)-(L2S1-L2S2)).
```

Callistoura: model comparisons of Full Model with submodels

```r
C.FM <- glmer(cbind(k.c, n-k.c) ~ Species + Loc + Season + Loc:Season
              + (1 | Site / Group),
              contrasts=list(Species=contr.sum, Loc=contr.sum, Season=contr.sum),
              family=binomial, data=Qarea.grp)
logLik(C.FM)
```

```
## 'log Lik.' -107.799 (df=9)
```

```r
# Null model: intercept only, neither fixed nor random effects.
C.null <- glm(cbind(k.c, n-k.c) ~ 1, family=binomial, data=Qarea.grp)
logLik(C.null)
```

```
## 'log Lik.' -137.852 (df=1)
```

```r
anova(C.FM, C.null, test="Chisq")
```

```
## Data: Qarea.grp
## Models:
## C.null: cbind(k.c, n - k.c) ~ 1
## C.FM: cbind(k.c, n - k.c) ~ Species + Loc + Season + Loc:Season + (1 |
## C.FM:     Site/Group)
##        Df   AIC    BIC  logLik deviance  Chisq Chi Df Pr(>Chisq)
## C.null  1 277.7 280.26 -137.85    275.7
```

```
## C.FM    9 233.6 256.58 -107.80    215.6 60.106      8  4.443e-10
```

```
# Highly significant Full-Null model comparison: fixed and/or random effects
# are found.

# Remove all random effects from FM
C.fixed <- glm(cbind(k.c, n-k.c) ~ Species + Loc + Season + Loc:Season,
               family=binomial, data=Qarea.grp)
logLik(C.fixed)
```

```
## 'log Lik.' -119.5333 (df=7)
```

```
anova(C.FM, C.fixed, test="Chisq")
```

```
## Data: Qarea.grp
## Models:
## C.fixed: cbind(k.c, n - k.c) ~ Species + Loc + Season + Loc:Season
## C.FM: cbind(k.c, n - k.c) ~ Species + Loc + Season + Loc:Season + (1 |
## C.FM:     Site/Group)
##         Df    AIC    BIC  logLik deviance  Chisq Chi Df Pr(>Chisq)
## C.fixed  7 253.07 270.94 -119.53   239.07
## C.FM     9 233.60 256.58 -107.80   215.60 23.469      2  8.014e-06
```

```
# Highly significant model comparison: random effects are found.

# Remove all fixed effects from FM
C.random <-  glmer(cbind(k.c, n-k.c)  ~ 1 + (1 | Site / Group),
                   family=binomial, data=Qarea.grp)
logLik(C.random)
```

```
## 'log Lik.' -112.1069 (df=3)
```

```
anova(C.FM, C.random, test="Chisq")
```

```
## Data: Qarea.grp
## Models:
## C.random: cbind(k.c, n - k.c) ~ 1 + (1 | Site/Group)
## C.FM: cbind(k.c, n - k.c) ~ Species + Loc + Season + Loc:Season + (1 |
## C.FM:     Site/Group)
##          Df    AIC    BIC  logLik deviance  Chisq Chi Df Pr(>Chisq)
## C.random  3 230.21 237.88 -112.11   224.21
## C.FM      9 233.60 256.58 -107.80   215.60 8.6158      6     0.1964
```

```
# Insignificant model comparison: no fixed effect found.

# Remove observation level random effect from FM (model without overdispersion)
C.site <- glmer(cbind(k.c, n-k.c) ~ Species + Loc + Season + Loc + Loc:Season +
                (1 | Site),  family=binomial, data=Qarea.grp)
logLik(C.site)
```

```
## 'log Lik.' -113.3738 (df=8)
```

```
anova(C.FM, C.site, test="Chisq")
```

```
## Data: Qarea.grp
```

```
## Models:
## C.site: cbind(k.c, n - k.c) ~ Species + Loc + Season + Loc + Loc:Season +
## C.site:     (1 | Site)
## C.FM: cbind(k.c, n - k.c) ~ Species + Loc + Season + Loc:Season + (1 |
## C.FM:     Site/Group)
##        Df    AIC    BIC  logLik deviance Chisq Chi Df Pr(>Chisq)
## C.site  8 242.75 263.18 -113.37   226.75
## C.FM    9 233.60 256.58 -107.80   215.60 11.15      1  0.0008405
```

```
# Highly significant model comparison: overdispersion is found.

# Remove Site random effect from FM
C.group <- glmer(cbind(k.c, n-k.c) ~ Species + Loc + Season + Loc:Season +
                 (1 | Group), family=binomial, data=Qarea.grp)
logLik(C.group)
```

```
## 'log Lik.' -109.851 (df=8)
```

```
anova(C.FM, C.group, test="Chisq")
```

```
## Data: Qarea.grp
## Models:
## C.group: cbind(k.c, n - k.c) ~ Species + Loc + Season + Loc:Season + (1 |
## C.group:     Group)
## C.FM: cbind(k.c, n - k.c) ~ Species + Loc + Season + Loc:Season + (1 |
## C.FM:     Site/Group)
##         Df   AIC    BIC  logLik deviance  Chisq Chi Df Pr(>Chisq)
## C.group  8 235.7 256.13 -109.85    219.7
## C.FM     9 233.6 256.58 -107.80    215.6 4.1039      1    0.04279
```

```
# Significant model comparison: site random effects are needed.

# Remove interaction Loc x Season from FM: main parameter of interest
C.additive <- glmer(cbind(k.c, n-k.c) ~ Species + Loc + Season +
                 (1 | Site / Group), family=binomial, data=Qarea.grp)
logLik(C.additive)
```

```
## 'log Lik.' -108.2846 (df=8)
```

```
anova(C.FM, C.additive, test="Chisq")
```

```
## Data: Qarea.grp
## Models:
## C.additive: cbind(k.c, n - k.c) ~ Species + Loc + Season + (1 | Site/Group)
## C.FM: cbind(k.c, n - k.c) ~ Species + Loc + Season + Loc:Season + (1 |
## C.FM:     Site/Group)
##            Df    AIC    BIC  logLik deviance  Chisq Chi Df Pr(>Chisq)
## C.additive  8 232.57 253.00 -108.28   216.57
## C.FM        9 233.60 256.58 -107.80   215.60 0.9711      1     0.3244
```

```
# Insignificant model comparison: no interaction found

summary(C.FM)
```

```
## Generalized linear mixed model fit by maximum likelihood (Laplace
##   Approximation) [glmerMod]
##  Family: binomial  ( logit )
## Formula: cbind(k.c, n - k.c) ~ Species + Loc + Season + Loc:Season + (1 |
##     Site/Group)
##    Data: Qarea.grp
##
##      AIC      BIC   logLik deviance df.resid
##    233.6    256.6   -107.8    215.6       86
##
## Scaled residuals:
##     Min      1Q  Median      3Q     Max
## -1.5876 -0.4822  0.4590  0.6025  1.2991
##
## Random effects:
##  Groups     Name        Variance Std.Dev.
##  Group:Site (Intercept) 1.1215   1.0590
##  Site       (Intercept) 0.5971   0.7727
## Number of obs: 95, groups:  Group:Site, 95; Site, 8
##
## Fixed effects:
##                Estimate Std. Error z value Pr(>|z|)
## (Intercept)      1.1472     0.4308   2.663  0.00775
## Species1        -1.3413     0.5761  -2.328  0.01990
## Species2        -0.8136     0.8344  -0.975  0.32957
## Species3         1.3204     0.6125   2.156  0.03110
## Loc1            -0.2842     0.4277  -0.664  0.50636
## Season1         -0.2399     0.2374  -1.010  0.31227
## Loc1:Season1     0.2381     0.2408   0.989  0.32265
##
## Correlation of Fixed Effects:
##             (Intr) Specs1 Specs2 Specs3 Loc1   Seasn1
## Species1    -0.350
## Species2     0.157 -0.241
## Species3     0.134 -0.364 -0.678
## Loc1         0.242 -0.022  0.397 -0.382
## Season1      0.167 -0.099  0.307 -0.111  0.024
## Loc1:Seasn1 -0.112  0.101 -0.356  0.293 -0.038 -0.359
```

Callistoura: R-squared for GLMM

Obtain R-squared for GLMM using the method proposed by Nakagawa and Schielzeth (2013) A general and simple method for obtaining R2 from generalized linear mixed-effects models, Methods in Ecology and Evolution 4, 133-142.

This method is available with function r.squaredGLMM() in MuMIn package.

```
r.squaredGLMM(C.FM)
```

```
##       R2m       R2c
## 0.1672960 0.2665625
```

Pseudo $R^2_{marginal} = 0.17$, $R^2_{cond} = 0.27$. These values are calculated with the observation-level random effect variance component (overdispersion) in the denominator.\ The marginal pseudo R^2_m tells that 17% of variation (on logit scale) is "explained" by the fixed part of model (species, location, season, location x season).\ The conditional pseudo R^2_c includes the variance component for sites into the numerator of the ratio. Now 27% of variation (on logit scale) is "explained".\ We must be *very* careful with interpretation of these values. F.i., the maximum attainable value for these pseudo R^2's is not equal to 1!

Analysis of Lemuricola infection prevalence

Again, we apply a GLMM for (aggregated) binomial data with fixed effects for season (early dry - early wet), location (East - West), and their interaction and control variable species, and random effects for Site and at observation-level.

Lemuricola: assumption checking for GLMM

The full model, described above, is fitted, for which we check assumptions.

```
L.FM <- glmer(cbind(k.l, n-k.l) ~ Species + Loc + Season + Loc:Season
              + (1 | Site / Group),
              contrasts=list(Species=contr.sum, Loc=contr.sum, Season=contr.sum),
              family=binomial, data=Qarea.grp)

par(mfrow=c(1,2))
res <- residuals(L.FM,  type="pearson")
lp  <- predict(L.FM)
qqnorm(res) # just to check for outliers, no normality assumed
plot(res ~ lp, data=Qarea.grp)
```

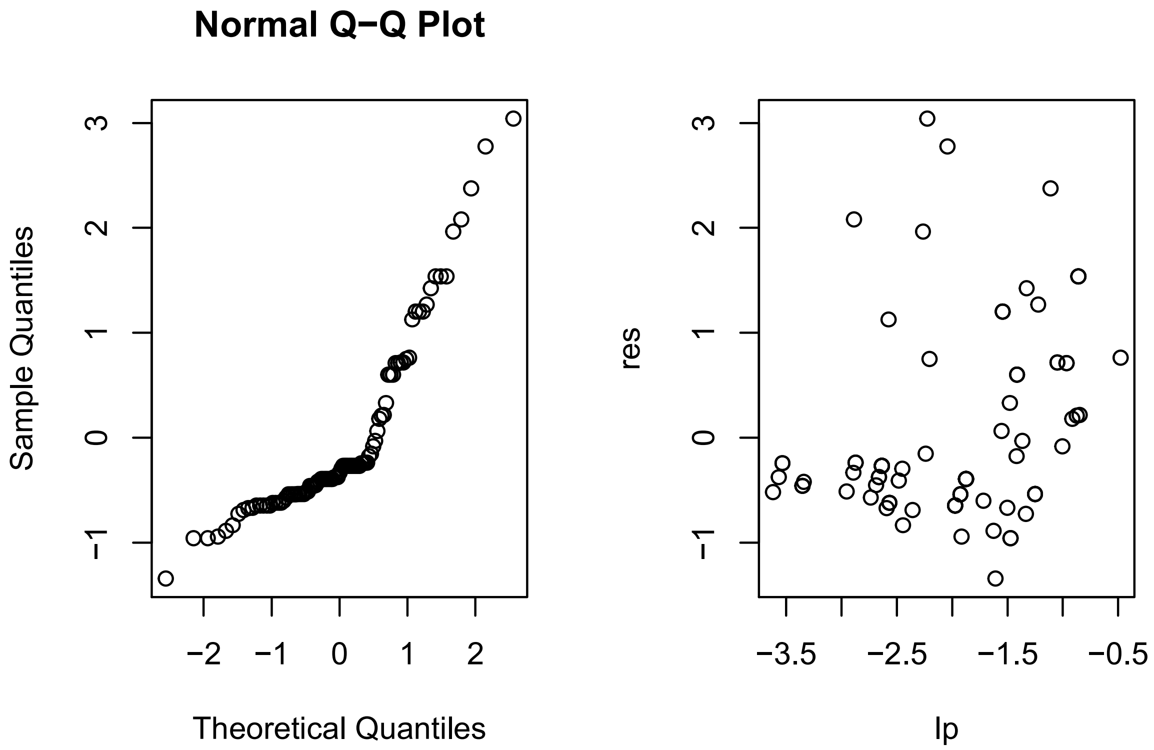


```r
par(mfrow=c(1,1))
# Neither outliers are found, nor extreme patterns in residual plot.

# Model check using residuald with DHARMa
set.seed(345)
simulationOutput <- simulateResiduals(fittedModel = L.FM, n=5000)
testUniformity(simulationOutput=simulationOutput)
```

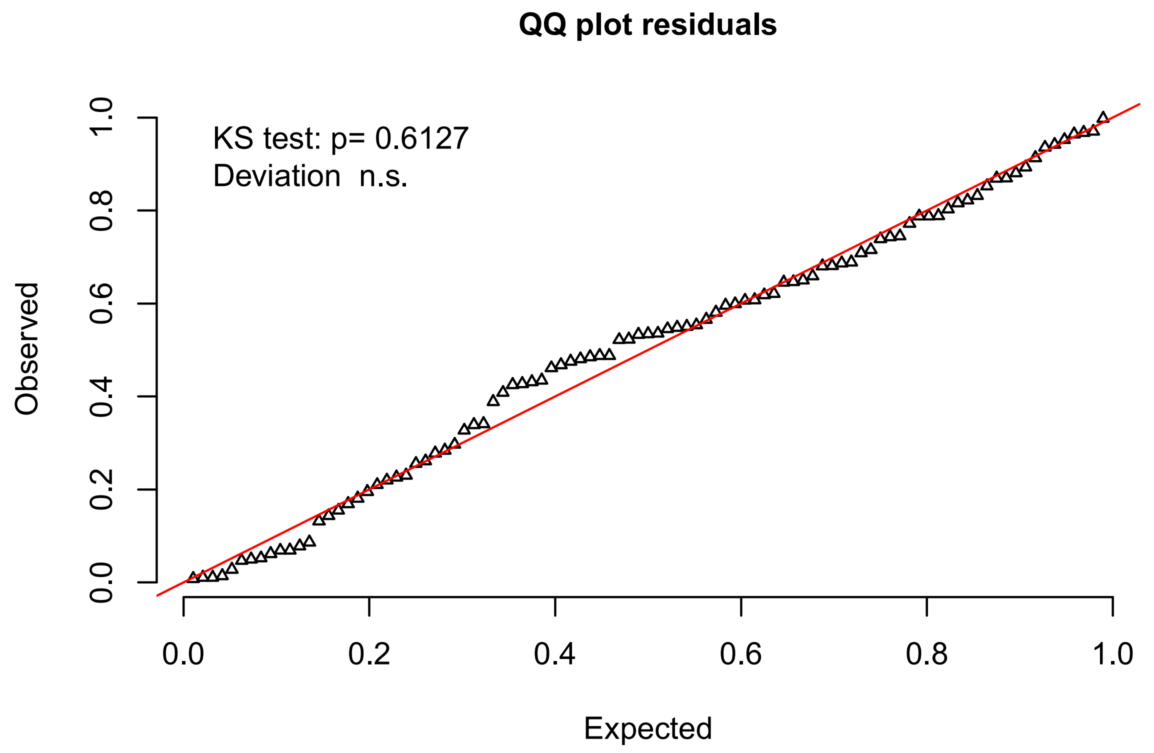


```
##
##  One-sample Kolmogorov-Smirnov test
##
## data:  simulationOutput$scaledResiduals
## D = 0.077832, p-value = 0.6127
## alternative hypothesis: two-sided
```

```
# We see no indications of problems.

# Again, to get a rough idea about leverage, we calculate leverages as would be
# used in GLM. Instead of the GLMM we fit an ordinary GLM:
L.lev <- hatvalues(glm(cbind(k.l, n-k.l) ~ Species + Season + Loc + Season:Loc,
                   family=binomial, data=Qarea.grp))
range(L.lev)
```

```
## [1] 0.01797561 0.32017273
```

```
(cutoff.lev <- 2*7/nrow(Qarea.grp)) # threshold for leverage in linear models
```

```
## [1] 0.1473684
```

```
sum(L.lev > cutoff.lev)
```

```
## [1] 9
```

```
# Largest leverage is 0.32, larger than the threshold 0.15 (=2*(7/95)), as would
# be used in LM; 9 observations have leverages larger than threshold.
Qarea.grp$n[L.lev > cutoff.lev]
```

```
## [1]  4  4 15 11  9 10  9  8 12
```

```
# Again, these are the observations with large social group sizes.

# To get a rough idea about collinearity among fixed factors, we calculate the
# generalized variance inflation factor, as would be used in LM using function
# vif from the car package. We don't know of a method for GLMM's.
vif(glm(cbind(k.l, n-k.l) ~ Species + Loc + Season + Loc:Season,
        family=binomial, data=Qarea.grp))
```

```
##                GVIF Df GVIF^(1/(2*Df))
## Species    4.225747  3        1.271503
## Loc        3.425137  1        1.850713
## Season     2.792547  1        1.671092
## Loc:Season 4.975361  1        2.230552
```

```
# Largest GVIF2 = 2.23 for Loc:Season, which is relatively small.

# We check for model stability using a script kindly made available by
# dr. Roger Mundry from the Max Planck Institute of Evolutionary Anthropology.
 L.FM2 <- glmer(cbind(k.l, n-k.l) ~ SpFulvusD + SpMacacoD + SpRubriventerD +
                 LocD + SeasonD + LSD +
                (1 | Site) + (1 | Group), family=binomial, data=Qarea.grp)

full.stab <- glmm.model.stab(model.res=L.FM2, para=F, data=Qarea.grp)
```

```
## [1] "please carefully evaluate whether the result makes sense, and if not, please contact me"
```

```
## fixed-effect model matrix is rank deficient so dropping 1 column / coefficient
```

```
round(full.stab$summary[, -1], 3)
```

```
##                          orig    min    max
## (Intercept)            -1.747 -1.955 -1.534
## SpFulvusD              -0.844 -1.153 -0.418
## SpMacacoD               0.598  0.101  1.186
## SpRubriventerD          0.848  0.587  1.325
## LocD                   -0.588 -0.888 -0.300
## SeasonD                 0.372  0.209  0.530
## LSD                    -0.042 -0.265  0.112
## Group@(Intercept)@NA    0.650  0.000  0.846
## Site@(Intercept)@NA     0.000  0.000  0.006
```

```
# Model results are most unstable for the interaction Location by Season
# (zero in range of estimates). All other variables are relatively stable,
# noticably the main effects of Location and Season.
```

Lemuricola: model comparisons of Full Model with submodels

```
L.FM <- glmer(cbind(k.l, n-k.l) ~ Species + Loc + Season + Loc:Season
              + (1 | Site / Group),
              contrasts=list(Species=contr.sum, Loc=contr.sum, Season=contr.sum),
```

```r
                family=binomial, data=Qarea.grp)
logLik(L.FM)
```

```
## 'log Lik.' -74.99733 (df=9)
```

```r
r.squaredGLMM(L.FM)
```

```
##       R2m       R2c
## 0.1211162 0.1211162
```

```r
# Remove both fixed and random effects from FM: intercept only
L.null <- glm(cbind(k.l, n-k.l) ~ 1, family=binomial, data=Qarea.grp)
logLik(L.null)
```

```
## 'log Lik.' -84.81756 (df=1)
```

```r
anova(L.FM, L.null, test="Chisq")
```

```
## Data: Qarea.grp
## Models:
## L.null: cbind(k.l, n - k.l) ~ 1
## L.FM: cbind(k.l, n - k.l) ~ Species + Loc + Season + Loc:Season + (1 |
## L.FM:     Site/Group)
##        Df    AIC    BIC  logLik deviance Chisq Chi Df Pr(>Chisq)
## L.null  1 171.63 174.19 -84.818   169.63
## L.FM    9 168.00 190.98 -74.997   150.00 19.64      8    0.01179
```

```r
# Significant model comparison: fixed and/or random effects found

# Remove all random effects from FM
L.fixed <- glm(cbind(k.l, n-k.l) ~ Species + Loc + Season + Loc:Season,
               family=binomial, data=Qarea.grp)
logLik(L.fixed)
```

```
## 'log Lik.' -75.69524 (df=7)
```

```r
anova(L.FM, L.fixed, test="Chisq")
```

```
## Data: Qarea.grp
## Models:
## L.fixed: cbind(k.l, n - k.l) ~ Species + Loc + Season + Loc:Season
## L.FM: cbind(k.l, n - k.l) ~ Species + Loc + Season + Loc:Season + (1 |
## L.FM:     Site/Group)
##         Df    AIC    BIC  logLik deviance  Chisq Chi Df Pr(>Chisq)
## L.fixed  7 165.39 183.27 -75.695   151.39
## L.FM     9 168.00 190.98 -74.997   150.00 1.3958      2     0.4976
```

```r
# Insignificant model model comparison: no random effect found

# Remove all fixed effects from FM
L.random <-  glmer(cbind(k.l, n-k.l)  ~ 1 + (1 | Site / Group),
                   family=binomial, data=Qarea.grp)
logLik(L.random)
```

```
## 'log Lik.' -82.18378 (df=3)
```

```r
anova(L.FM, L.random, test="Chisq")
```

```
## Data: Qarea.grp
## Models:
## L.random: cbind(k.l, n - k.l) ~ 1 + (1 | Site/Group)
## L.FM: cbind(k.l, n - k.l) ~ Species + Loc + Season + Loc:Season + (1 |
## L.FM:     Site/Group)
##          Df    AIC    BIC  logLik deviance  Chisq Chi Df Pr(>Chisq)
## L.random  3 170.37 178.03 -82.184   164.37
## L.FM      9 168.00 190.98 -74.997   150.00 14.373      6    0.02574
```

```r
# Significant model comparison: fixed effects found

# Remove interaction Loc:Season from FM
L.Sp.random <- glmer(cbind(k.l, n-k.l) ~ Species + (1 | Site / Group),
                    contrasts=list(Species=contr.sum),
                    family=binomial, data=Qarea.grp)
logLik(L.Sp.random)
```

```
## 'log Lik.' -79.2021 (df=6)
```

```r
anova(L.FM, L.Sp.random, test="Chisq")
```

```
## Data: Qarea.grp
## Models:
## L.Sp.random: cbind(k.l, n - k.l) ~ Species + (1 | Site/Group)
## L.FM: cbind(k.l, n - k.l) ~ Species + Loc + Season + Loc:Season + (1 |
## L.FM:     Site/Group)
##             Df   AIC    BIC  logLik deviance  Chisq Chi Df Pr(>Chisq)
## L.Sp.random  6 170.4 185.73 -79.202    158.4
## L.FM         9 168.0 190.98 -74.997    150.0 8.4095      3    0.03826
```

```r
# Significant model comparison: fixed effects of Loc, Season, and/or interaction

# Remove interaction Loc:Season from FM
L.additive <- glmer(cbind(k.l, n-k.l) ~ Species + Season + Loc
                    + (1 | Site / Group),
                    contrasts=list(Species=contr.sum, Season=contr.sum, Loc=contr.sum),
                    family=binomial, data=Qarea.grp)
logLik(L.additive)
```

```
## 'log Lik.' -75.0174 (df=8)
```

```r
anova(L.FM, L.additive, test="Chisq")
```

```
## Data: Qarea.grp
## Models:
## L.additive: cbind(k.l, n - k.l) ~ Species + Season + Loc + (1 | Site/Group)
## L.FM: cbind(k.l, n - k.l) ~ Species + Loc + Season + Loc:Season + (1 |
## L.FM:     Site/Group)
##            Df    AIC    BIC  logLik deviance  Chisq Chi Df Pr(>Chisq)
## L.additive  8 166.03 186.47 -75.017   150.03
## L.FM        9 168.00 190.98 -74.997   150.00 0.0401      1     0.8412
```

```r
# Insignificant model comparison: no interaction Loc:Season found;
# this parameter was unstable in stability analysis.

# Remove main effect location from FM
L.SpS <- glmer(cbind(k.l, n-k.l) ~ Species + SeasonD + LSD + (1 | Site / Group),
               contrasts=list(Species=contr.sum), family=binomial, data=Qarea.grp)
logLik(L.SpS)
```

```
## 'log Lik.' -77.30905 (df=8)
```

```r
anova(L.FM, L.SpS, test="Chisq")
```

```
## Data: Qarea.grp
## Models:
## L.SpS: cbind(k.l, n - k.l) ~ Species + SeasonD + LSD + (1 | Site/Group)
## L.FM: cbind(k.l, n - k.l) ~ Species + Loc + Season + Loc:Season + (1 |
## L.FM:     Site/Group)
##       Df    AIC    BIC  logLik deviance  Chisq Chi Df Pr(>Chisq)
## L.SpS  8 170.62 191.05 -77.309   154.62
## L.FM   9 168.00 190.98 -74.997   150.00 4.6234      1    0.03154
```

```r
# Significant model comparison: main effect location found

# Remove main effect season from FM
L.SpL <- glmer(cbind(k.l, n-k.l) ~ Species + LocD + LSD + (1 | Site / Group),
               contrasts=list(Species=contr.sum), family=binomial, data=Qarea.grp)
logLik(L.SpL)
```

```
## 'log Lik.' -76.59165 (df=8)
```

```r
anova(L.FM, L.SpL, test="Chisq")
```

```
## Data: Qarea.grp
## Models:
## L.SpL: cbind(k.l, n - k.l) ~ Species + LocD + LSD + (1 | Site/Group)
## L.FM: cbind(k.l, n - k.l) ~ Species + Loc + Season + Loc:Season + (1 |
## L.FM:     Site/Group)
##       Df    AIC    BIC  logLik deviance  Chisq Chi Df Pr(>Chisq)
## L.SpL  8 169.18 189.61 -76.592   153.18
## L.FM   9 168.00 190.98 -74.997   150.00 3.1886      1    0.07415
```

```r
# Insignificant model comparison: no season main effect found
summary(L.FM)
```

```
## Generalized linear mixed model fit by maximum likelihood (Laplace
##   Approximation) [glmerMod]
##  Family: binomial  ( logit )
## Formula: cbind(k.l, n - k.l) ~ Species + Loc + Season + Loc:Season + (1 |
##     Site/Group)
##    Data: Qarea.grp
##
##      AIC      BIC   logLik deviance df.resid
##      168      191      -75      150       86
```

```
##
## Scaled residuals:
##     Min      1Q  Median      3Q     Max
## -1.3417 -0.5400 -0.3328  0.2752  3.0422
##
## Random effects:
##  Groups     Name        Variance  Std.Dev.
##  Group:Site (Intercept) 4.231e-01 0.6504275
##  Site       (Intercept) 1.003e-08 0.0001001
## Number of obs: 95, groups:  Group:Site, 95; Site, 8
##
## Fixed effects:
##              Estimate Std. Error z value Pr(>|z|)
## (Intercept)   -1.7470     0.2458  -7.108 1.18e-12
## Species1      -0.8439     0.3445  -2.450   0.0143
## Species2       0.5979     0.5150   1.161   0.2456
## Species3       0.8478     0.4896   1.731   0.0834
## Loc1          -0.5884     0.2907  -2.024   0.0430
## Season1        0.3721     0.2176   1.710   0.0873
## Loc1:Season1  -0.0418     0.2086  -0.200   0.8412
##
## Correlation of Fixed Effects:
##             (Intr) Specs1 Specs2 Specs3 Loc1   Seasn1
## Species1     0.026
## Species2     0.311 -0.313
## Species3    -0.330 -0.307 -0.587
## Loc1         0.370  0.224  0.462 -0.755
## Season1      0.039 -0.212  0.354  0.015 -0.013
## Loc1:Seasn1 -0.175  0.142 -0.412  0.265 -0.143 -0.005
```

```r
# Remove main effect species from FM
L.LS <- glmer(cbind(k.l, n-k.l) ~ Loc + Season + Loc:Season + (1 | Site / Group),
             family=binomial, data=Qarea.grp)
logLik(L.LS)
```

```
## 'log Lik.' -80.51072 (df=6)
```

```r
anova(L.FM, L.LS, test="Chisq")
```

```
## Data: Qarea.grp
## Models:
## L.LS: cbind(k.l, n - k.l) ~ Loc + Season + Loc:Season + (1 | Site/Group)
## L.FM: cbind(k.l, n - k.l) ~ Species + Loc + Season + Loc:Season + (1 |
## L.FM:     Site/Group)
##      Df    AIC    BIC  logLik deviance  Chisq Chi Df Pr(>Chisq)
## L.LS  6 173.02 188.34 -80.511   161.02
## L.FM  9 168.00 190.98 -74.997   150.00 11.027      3    0.01158
```

```r
# Significant model comparison: species main effect found

summary(L.FM)
```

```
## Generalized linear mixed model fit by maximum likelihood (Laplace
```

```
##   Approximation) [glmerMod]
##  Family: binomial  ( logit )
## Formula: cbind(k.l, n - k.l) ~ Species + Loc + Season + Loc:Season + (1 |
##     Site/Group)
##    Data: Qarea.grp
##
##      AIC      BIC   logLik deviance df.resid
##      168      191      -75      150       86
##
## Scaled residuals:
##     Min      1Q  Median      3Q     Max
## -1.3417 -0.5400 -0.3328  0.2752  3.0422
##
## Random effects:
##  Groups     Name        Variance  Std.Dev.
##  Group:Site (Intercept) 4.231e-01 0.6504275
##  Site       (Intercept) 1.003e-08 0.0001001
## Number of obs: 95, groups:  Group:Site, 95; Site, 8
##
## Fixed effects:
##              Estimate Std. Error z value Pr(>|z|)
## (Intercept)   -1.7470     0.2458  -7.108 1.18e-12
## Species1      -0.8439     0.3445  -2.450   0.0143
## Species2       0.5979     0.5150   1.161   0.2456
## Species3       0.8478     0.4896   1.731   0.0834
## Loc1          -0.5884     0.2907  -2.024   0.0430
## Season1        0.3721     0.2176   1.710   0.0873
## Loc1:Season1  -0.0418     0.2086  -0.200   0.8412
##
## Correlation of Fixed Effects:
##             (Intr) Specs1 Specs2 Specs3 Loc1   Seasn1
## Species1     0.026
## Species2     0.311 -0.313
## Species3    -0.330 -0.307 -0.587
## Loc1         0.370  0.224  0.462 -0.755
## Season1      0.039 -0.212  0.354  0.015 -0.013
## Loc1:Seasn1 -0.175  0.142 -0.412  0.265 -0.143 -0.005
```

Lemuricola: mean prevalences by location.

```
emmeans(L.FM, ~ Loc)
```

```
## NOTE: Results may be misleading due to involvement in interactions
```

```
##  Loc     emmean        SE  df asymp.LCL  asymp.UCL
##  East -2.335492 0.4447463 Inf -3.207178 -1.4638051
##  West -1.158617 0.3034112 Inf -1.753292 -0.5639422
##
## Results are averaged over the levels of: Species, Season
## Results are given on the logit (not the response) scale.
```

```
## Confidence level used: 0.95
```

```r
vc <- VarCorr(L.FM)
varcomps <- as.data.frame(vc)[,4]
s2u <- sum(varcomps)

co <- (15/16)*pi/sqrt(3)

# shrinkage factor:
lambda <- 1 / sqrt(s2u/(co^2) + 1)

(emm.L <- summary(emmeans(L.FM, ~ Loc)))
```

```
## NOTE: Results may be misleading due to involvement in interactions
```

```
##  Loc     emmean        SE  df asymp.LCL  asymp.UCL
##  East -2.335492 0.4447463 Inf -3.207178 -1.4638051
##  West -1.158617 0.3034112 Inf -1.753292 -0.5639422
##
## Results are averaged over the levels of: Species, Season
## Results are given on the logit (not the response) scale.
## Confidence level used: 0.95
```

```r
emm.L.back.med <- 1/(1+exp(-emm.L[,2]))
emm.L.lo.med   <- 1/(1+exp(-emm.L[,5]))
emm.L.up.med   <- 1/(1+exp(-emm.L[,6]))

emm.L.back.mean <- 1/(1+exp(-lambda*emm.L[,2]))
emm.L.lo.mean   <- 1/(1+exp(-lambda*emm.L[,5]))
emm.L.up.mean   <- 1/(1+exp(-lambda*emm.L[,6]))



emm.L <- data.frame(emm.L[,1],
                    emm.L.lo.med,  emm.L.back.med,  emm.L.up.med,
                    emm.L.lo.mean, emm.L.back.mean, emm.L.up.mean)

names(emm.L) <- c("Location",
                  "lo 95 med p", "backtr.med p","up 95 median p",
                  "lo 95 mean p", "backtr.mean p","up 95 mean p")
data.frame(Location=emm.L[,1:1], round(emm.L[,2:7],3))
```

```
##   Location lo.95.med.p backtr.med.p up.95.median.p lo.95.mean.p
## 1     East       0.039        0.088          0.188        0.048
## 2     West       0.148        0.239          0.363        0.163
##   backtr.mean.p up.95.mean.p
## 1         0.101        0.203
## 2         0.253        0.371
```

Lemuricola: R-squared for GLMM

```r
r.squaredGLMM(L.FM)
```

```
##       R2m       R2c
## 0.1211162 0.1211162
```

The marginal pseudo R_m^2 is 12%. The variance compnenent for sites is so small that inclusion into the numerator, leading to R_c^2, still leaves its value at 12%.

Coinfection of Callistoura and Lemuricola

To check whether coinfection with Lemuricola is related to change in Callistoura infection prevalence we expand the original full model for Callistoura to include an indicator variable for Lemuricola infection.

```
CL.FM <- glmer(cbind(CalPrev, 1-CalPrev) ~ Species + Season + Loc + Season:Loc + LemPrev +
                 (1 | Site / Group), family=binomial, data=Qarea)
drop1(CL.FM, test="Chisq")
```

```
## Single term deletions
##
## Model:
## cbind(CalPrev, 1 - CalPrev) ~ Species + Season + Loc + Season:Loc +
##     LemPrev + (1 | Site/Group)
##             Df    AIC    LRT Pr(Chi)
## <none>         387.37
## Species      3 388.31 6.9441  0.0737
## LemPrev      1 386.18 0.8147  0.3667
## Season:Loc   1 386.31 0.9377  0.3329
```

We find no evidence of change in infection prevalence for Callistoura comparing Lemuricola infected versus uninfected individuals (P=0.33).

Callistoura and Lemuricola prevalence in Eulemurs: comparison of more and less disturbed sites in Ranomafana

Gerrit Gort and Iris de Winter

16-07-2018

Read parasites data

We select from the database the data from site Ranomafana, more specifically the locations of Tala (disturbed) and Valo and Vato (less disturbed). The data from undisturbed specific locations Valo and Vato are combined.

```
parasites <- read_excel("2017_11_20_Supplementary Dataset S1.xls",
                        sheet="Supplementary Dataset S1", na="U")
Qdist <- data.frame(parasites[
          parasites$Site=="Ranomafana",
          c("Site","SpecificLoc","Species","Group","Season",
            "CalPrev","LemPrev")])
Qdist <- Qdist[Qdist$SpecificLoc %in%  c("Tala","Valo","Vato"),]

Qdist$SpLoc <- factor(Qdist$SpecificLoc)
Qdist$Dist <- Qdist$SpLoc
levels(Qdist$Dist) <- c("Y","N","N")

Qdist$Season <- factor(Qdist$Season)
levels(Qdist$Season) <- c("Dry", "Wet")
Qdist$Site   <- factor(Qdist$Site)
Qdist$Group  <- factor(Qdist$Group)

Qdist <- droplevels(Qdist)

Qdist <- data.frame(Qdist[,c("Site","SpLoc","Dist","Season", "Species",
                             "Group", "CalPrev", "LemPrev")])
head(Qdist)
```

```
##         Site SpLoc Dist Season     Species Group CalPrev LemPrev
## 1 Ranomafana  Tala    Y    Wet Rubriventer     1       1       1
## 2 Ranomafana  Tala    Y    Wet Rubriventer     1       1       0
## 3 Ranomafana  Tala    Y    Wet Rubriventer     5       0       0
## 4 Ranomafana  Tala    Y    Wet Rubriventer     3       0       0
## 5 Ranomafana  Tala    Y    Wet Rubriventer     6       1       0
## 6 Ranomafana  Tala    Y    Wet Rubriventer     5       0       1
```

Aggregate data to level of social group.

Within subset 43 social groups, with number of lemurs ranging from 1 to 7. Average group size is 2.4, median group is 2.

```r
Qdist.grp <- summaryBy(CalPrev + LemPrev ~
              Season + SpLoc + Dist + Species + Group, data=Qdist, FUN=c(sum, length))

Qdist.grp <- Qdist.grp[,-9]
names(Qdist.grp)[6:8] <- c("k.c","k.l","n")
head(Qdist.grp)
```

```
##   Season SpLoc Dist     Species Group k.c k.l n
## 1    Dry  Tala    Y Rubriventer    44   2   1 2
## 2    Dry  Tala    Y Rubriventer    45   0   0 1
## 3    Dry  Tala    Y Rubriventer    46   1   1 1
## 4    Dry  Tala    Y Rubriventer    47   1   1 1
## 5    Dry  Tala    Y Rubriventer    48   1   1 1
## 6    Dry  Tala    Y Rubriventer    54   1   0 1
```

Descriptive statistics: frequency tables

```r
# summary statistics for group sizes
length(Qdist.grp$n)
```

```
## [1] 43
```

```r
summary(Qdist.grp$n)
```

```
##    Min. 1st Qu.  Median    Mean 3rd Qu.    Max.
##   1.000   1.000   2.000   2.395   3.000   7.000
```

```r
# frequency table of lemur individuals by disturbance (specific location) and species
addmargins(xtabs(n ~ Species + Dist, data=Qdist.grp))
```

```
##              Dist
## Species         Y   N Sum
##   Rubriventer  30  29  59
##   Rufifrons     8  36  44
##   Sum          38  65 103
```

```r
# frequency table of lemurs by disturbance (specific location), season and species
ftable(xtabs(n ~  Species + Season + Dist, data=Qdist.grp))
```

```
##                    Dist  Y  N
## Species     Season
## Rubriventer Dry          8 12
##             Wet         22 17
## Rufifrons   Dry          8 13
##             Wet          0 23
```

```r
# No Rufifrons in disturbed area (Tala) in early wet season

# frequency table of lemurs by disturban (specific location), season and Callistoura
# infection
ftable(xtabs( ~  Dist + Season + CalPrev, data=Qdist))
```

```
##              CalPrev  0  1
## Dist Season
## Y    Dry              6 10
##      Wet             10 12
## N    Dry              2 23
##      Wet             12 28
```

```
# frequency table of lemurs by disturbance (specific location), season and Lemuricola
# infection
ftable(xtabs( ~  Dist + Season + LemPrev, data=Qdist))
```

```
##              LemPrev  0  1
## Dist Season
## Y    Dry             10  6
##      Wet             15  7
## N    Dry             24  1
##      Wet             40  0
```

Main summary statistics:

- 43 social groups with group size range 1-; average group size 2.4, mean size 2.
- 103 individual lemur observations
- 2 Eulemur species: E. rubriventer and E. rufifrons
- no Rufifrons lemurs were observed in the disturbed area (Tala) during wet season
- Callistoura prevalence: 73 out of 103 (71%); Lemuricola prevalence: 14 out of 103 (14%)

Analysis of Callistoura infection prevalence for disturbance

For group level data an ordinary GLM with binomial distribution and logit link function is used, possibly with overdispersion. The GLM comprises fixed effects for disturbance (No - Yes), season (early dry - early wet), disturbance by season interaction, and control variable species.

Callistoura: binomial overdispersion

The full model, described above, is fitted and assumptions are checked. First we check for overdispersion. If present, we use William's method as available in R package dispmod, because the group sizes are quite different: groups have 1 - 7 individuals with an average of 2.4. Parameter phi is estimated: $Var(y_i) = m_i * \theta * (1 - \theta) * (1 + (m_i - 1) * \phi)$. A value $\phi > 0$ indicates binomial overdispersion.

```
# First focus on overdispersion.
DC.FM  <- glm(cbind(k.c, n-k.c) ~ Species + Dist + Season + Dist:Season,
              contrasts=list(Species=contr.sum, Dist=contr.sum, Season=contr.sum),
              family=binomial, data=Qdist.grp)

(res.dev <- deviance(DC.FM))
```

```
## [1] 67.39969
```

```r
(res.df <- df.residual(DC.FM))
```

```
## [1] 38
```

```r
1-pchisq(res.dev, res.df)
```

```
## [1] 0.002301086
```

```r
# Overdispersion: residual deviance is substantially larger than residual df.

# William's method for handling overdispersion
DCWilliams.FM <- glm.binomial.disp(DC.FM, maxit=100, verbose=FALSE)
DCWilliams.FM$dispersion
```

```
## [1] 0.4666597
```

```r
Qdist.grp$w <- DCWilliams.FM$disp.weights # weights
logLik(DCWilliams.FM)
```

```
## 'log Lik.' -24.73509 (df=5)
```

Overdispersion parameter phi is 0.47, indicating slight binomial overdispersion. We modify the glm fit using Williams's method. This amounts to the use of weights w.

Callistoura: continued assumption checking for overdispersed GLM

```r
DC.W.FM <- glm(cbind(k.c, n-k.c) ~ Species + Dist + Season + Dist:Season,
              contrasts=list(Species=contr.sum, Dist=contr.sum, Season=contr.sum),
              weights=w,
              family=binomial, data=Qdist.grp)
logLik(DC.W.FM)
```

```
## 'log Lik.' -24.73509 (df=5)
```

```r
par(mfrow=c(2,2), mar=c(3,3,2,0))
plot(DC.W.FM)
```

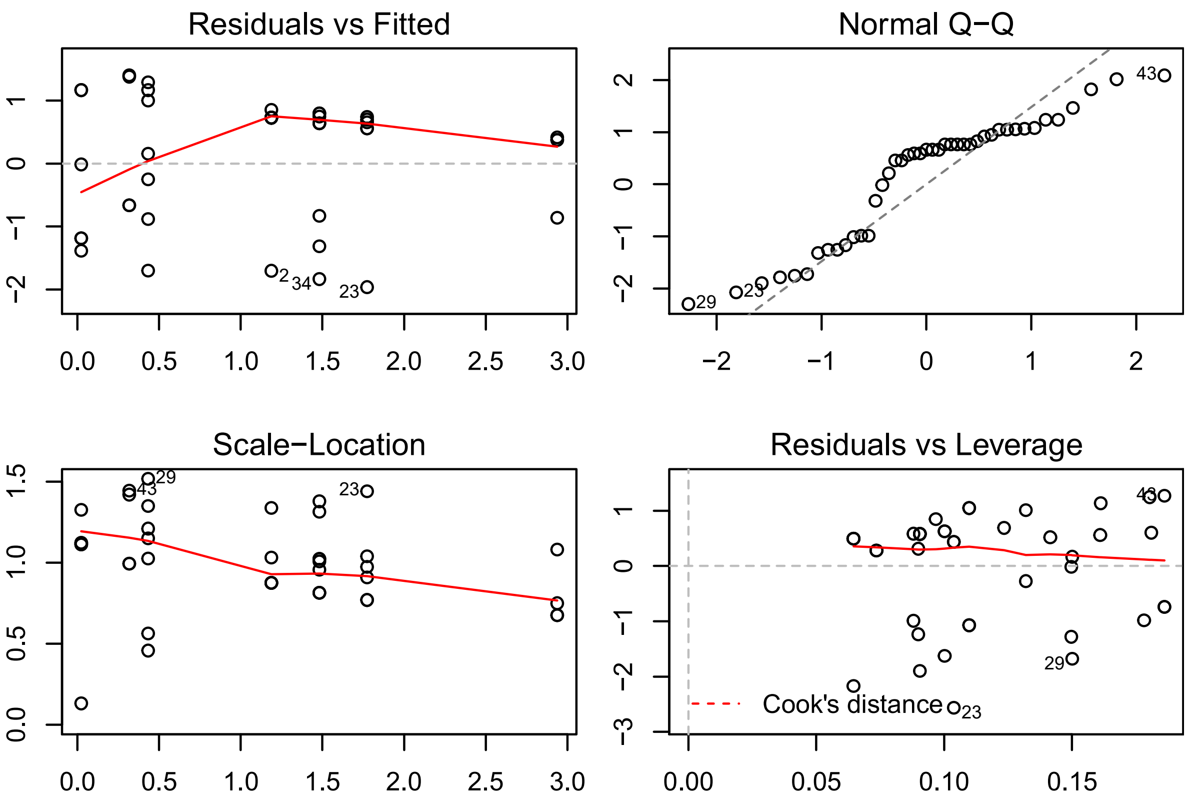


```
par(mfrow=c(1,1))

# Check GLM leverages.
C.lev <- hatvalues(DC.W.FM)
range(C.lev)
```

```
## [1] 0.06456488 0.18610788
```

```
(cutoff.lev <- 2*5/nrow(Qdist.grp))
```

```
## [1] 0.2325581
```

```
sum(C.lev > cutoff.lev)
```

```
## [1] 0
```

```
# Largest leverage is 0.19, smaller than the threshold 0.23 (=2*(5/43)).
# Hence, no observations with large potential influence.

# Collinearity of explanatory factors: variance inflation factors.
vif(DC.W.FM)
```

```
##      Species        Dist      Season Dist:Season
##     1.364935    1.332877    1.362735    1.108564
```

```
# Largest VIF = 1.365 for Species, which is small. Collinearity is no problem here.

# Although all leverages are rather small, we check DFbetas:
C.dfbetas <- dfbetas(DC.W.FM)
```

```r
par(mfrow=c(1,5), mar=c(4,3,2,0))
hist(C.dfbetas[,1], main="overall mean", xlab="dfbetas")
hist(C.dfbetas[,2], main="half species diff", xlab="dfbetas")
hist(C.dfbetas[,3], main="half disturbance diff", xlab="dfbetas")
hist(C.dfbetas[,4], main="half season diff", xlab="dfbetas")
hist(C.dfbetas[,5], main="interaction dist-season", xlab="dfbetas")
```

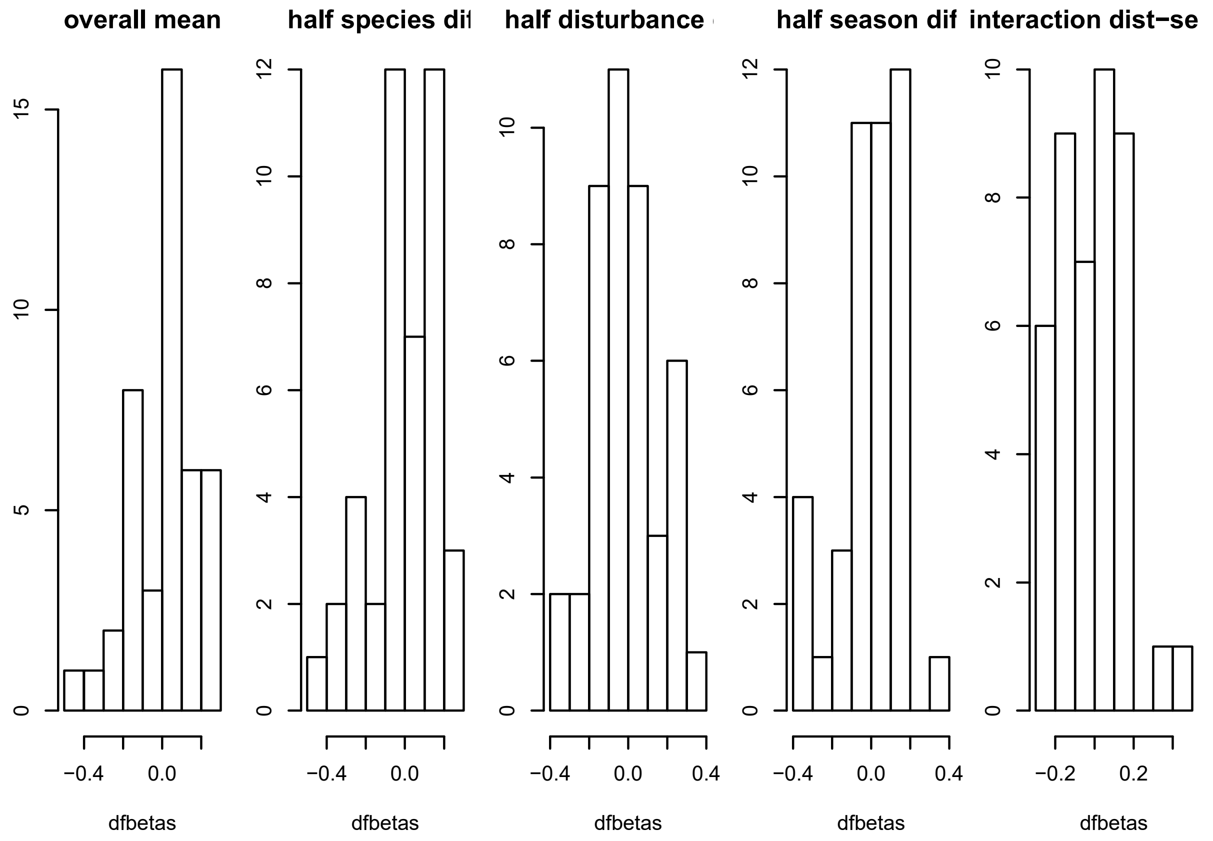


```r
par(mfrow=c(1,1))
# No dfbeta comes close to value 2. It appears there are no
# observations with unduely large influence on individual parameters

# Finally, an influencePlot, plotting studentized residuals versus leverage with bubble size defi
influencePlot(DC.W.FM)
```

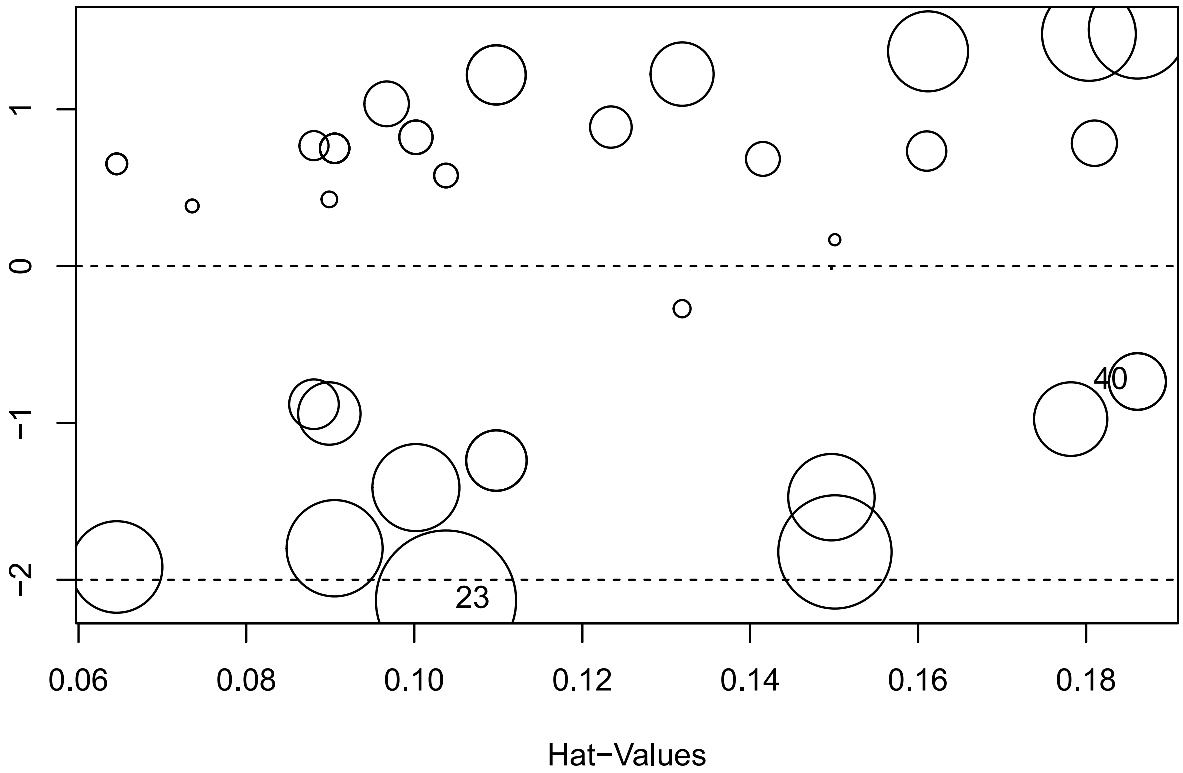


```
##        StudRes       Hat      CookD
## 23 -2.1312372 0.1037645 0.15218347
## 40 -0.7358846 0.1861079 0.02499475
```

```
# Our conclusion is that, although some observations are more influential than others,
# no observation has an overwhelmingly large impact. For sure P-values will change
# slightly by removal of observations, but differences are relatively small.
# Still, we need to be very careful with drawing strong conclusions about the data,
# if only because effects of disturbance and subsite are confounded in this dataset.
```

Callistoura: model comparisons of Full Model with submodels

We now perform model comparisons, always the full model with a submodel. The weights, according to William's method for overdispersion, estimated from the full model, will be used all the time.

```
DC.W.FM  <- glm(cbind(k.c, n-k.c) ~ Species + Dist + Season + Dist:Season,
              contrasts=list(Species=contr.sum, Dist=contr.sum, Season=contr.sum),
              weights=w, family=binomial, data=Qdist.grp)
logLik(DC.W.FM)
```

```
## 'log Lik.' -24.73509 (df=5)
```

```
# Null model: intercept only, no fixed effects
DC.W.null  <- glm(cbind(k.c, n-k.c) ~ 1, weights=w, family=binomial, data=Qdist.grp)
logLik(DC.W.null)
```

```
## 'log Lik.' -27.83475 (df=1)
```

```r
anova(DC.W.FM, DC.W.null, test="Chisq")
```

```
## Analysis of Deviance Table
##
## Model 1: cbind(k.c, n - k.c) ~ Species + Dist + Season + Dist:Season
## Model 2: cbind(k.c, n - k.c) ~ 1
##   Resid. Df Resid. Dev Df Deviance Pr(>Chi)
## 1        38     42.303
## 2        42     48.502 -4  -6.1993   0.1847
```

```r
# Insignificant model comparison: no effects are found.

# Main question is about differences in infection prevalence between disturbed and
# undisturbed areas. Regardless the outcome of the omnibus model, we test the main
# effect of disturbance within the full model.
# To test for main effects in presence of interaction effects, sum-to-zero
# restricted parameterization are needed.
Qdist.grp$dSp <-
  -1*(Qdist.grp$Species=="Rubriventer") + 1*(Qdist.grp$Species=="Rufifrons")
Qdist.grp$dD  <- -1*(Qdist.grp$Dist=="Y") + 1*(Qdist.grp$Dist=="N")
Qdist.grp$dS  <- -1*(Qdist.grp$Season=="Dry") + 1*(Qdist.grp$Season=="Wet")
Qdist.grp$dDS <- Qdist.grp$dD * Qdist.grp$dS

DC.W.FM2  <- glm(cbind(k.c, n-k.c) ~ dSp + dD + dS + dDS, weights=w,
                family=binomial, data=Qdist.grp)
logLik(DC.W.FM2) # same as model DCw.FM fitted earlier
```

```
## 'log Lik.' -24.73509 (df=5)
```

```r
# Remove main effect of disturbance
DC.W.SpSDS  <- glm(cbind(k.c, n-k.c) ~ dSp + dS + dDS, weights=w,
                  family=binomial, data=Qdist.grp)
logLik(DC.W.SpSDS)
```

```
## 'log Lik.' -26.80309 (df=4)
```

```r
anova(DC.W.FM2, DC.W.SpSDS, test="Chisq")
```

```
## Analysis of Deviance Table
##
## Model 1: cbind(k.c, n - k.c) ~ dSp + dD + dS + dDS
## Model 2: cbind(k.c, n - k.c) ~ dSp + dS + dDS
##   Resid. Df Resid. Dev Df Deviance Pr(>Chi)
## 1        38     42.303
## 2        39     46.439 -1   -4.136  0.04198
```

```r
# Significant model comparison: main effect of disturbance found

summary(DC.W.FM)
```

```
##
## Call:
## glm(formula = cbind(k.c, n - k.c) ~ Species + Dist + Season +
```

```
##     Dist:Season, family = binomial, data = Qdist.grp, weights = w,
##     contrasts = list(Species = contr.sum, Dist = contr.sum, Season = contr.sum))
##
## Deviance Residuals:
##     Min       1Q   Median       3Q      Max
## -1.9647  -0.7466   0.6399   0.7434   1.4031
##
## Coefficients:
##               Estimate Std. Error z value Pr(>|z|)
## (Intercept)     0.9280     0.3400   2.729  0.00635
## Species1        0.5821     0.3700   1.573  0.11567
## Dist1          -0.6997     0.3631  -1.927  0.05400
## Season1         0.5524     0.3713   1.488  0.13677
## Dist1:Season1  -0.1754     0.3430  -0.511  0.60920
##
## (Dispersion parameter for binomial family taken to be 1)
##
##     Null deviance: 48.502  on 42  degrees of freedom
## Residual deviance: 42.303  on 38  degrees of freedom
## AIC: 59.47
##
## Number of Fisher Scoring iterations: 4
```

Callistoura: mean prevalences by disturbance

Now the backtransformed predicted means for disturbance.

```
(emm.dist.cal <- summary(emmeans(DC.FM,"Dist")))
```

```
## NOTE: Results may be misleading due to involvement in interactions
```

```
##  Dist    emmean        SE  df  asymp.LCL asymp.UCL
##  Y    0.1276556 0.3631156 Inf -0.5840380 0.8393492
##  N    1.7550547 0.4247421 Inf  0.9225754 2.5875340
##
## Results are averaged over the levels of: Species, Season
## Results are given on the logit (not the response) scale.
## Confidence level used: 0.95
```

```
emm.dist.cal.back <- 1/(1+exp(-emm.dist.cal[,2]))
emm.dist.cal.lo   <- 1/(1+exp(-emm.dist.cal[,5]))
emm.dist.cal.up   <- 1/(1+exp(-emm.dist.cal[,6]))
data.frame(dist=emm.dist.cal[,1], emm.dist.cal.lo, emm.dist.cal.back, emm.dist.cal.up)
```

```
##   dist emm.dist.cal.lo emm.dist.cal.back emm.dist.cal.up
## 1    Y       0.3580040         0.5318706       0.6983281
## 2    N       0.7155666         0.8525892       0.9300550
```

Callistoura: Rsquared

```
r.squaredGLMM(DC.W.FM)
```

```
##        R2m       R2c
## 0.1670663 0.1670663
```

The pseudo R_m^2 is 17%. There are no random effects in this glm.

Analysis of Lemuricola infection prevalence for disturbance

Again, for group level data an ordinary GLM with binomial distribution and logit link function is used, possibly with overdispersion. The GLM comprises fixed effects for disturbance (No - Yes), season (early dry - early wet), disturbance by season interaction, and control variable species.\ \ The number of events of Lemuricola infection is small: only 14 events (out of 103 cases) our counted. There are no events in less disturbed area during the wet season, and only one during the dry season (also see frequency tables shown earlier):

```
ftable(xtabs( ~  Dist + Season + LemPrev, data=Qdist))
```

```
##              LemPrev  0  1
## Dist Season
## Y    Dry             10  6
##      Wet             15  7
## N    Dry             24  1
##      Wet             40  0
```

Because of the small number of events, the opportunities for modeling are limited. Sometimes in logistic regression a rule of thumb is used, expressing that at least 10 events per regressor are needed. This rule of thumb may be relaxed (see e.g. Relaxing the Rule of Ten Events per Variable in Logistic and Cox Regression, Eric Vittinghoff Charles E. McCulloch, American Journal of Epidemiology, Volume 165, Issue 6, 15 March 2007, Pages 710-718, https://doi.org/10.1093/aje/kwk052).\ \ We will fit a binomial glm (possibly with overdispersion) with effects for species, disturbance and season, but no interactions. Compared to the analysis of Callistoura prevalence we remove the interaction of disturbance and season (which was not important for Callistoura prevalence).

Lemuricola: binomial overdispersion

First check whether overdispersion is present.

```
DL.FM  <- glm(cbind(k.l, n-k.l) ~ Species + Dist + Season,
              contrasts=list(Species=contr.sum, Dist=contr.sum, Season=contr.sum),
              family=binomial, data=Qdist.grp)
(res.dev <- deviance(DL.FM))
```

```
## [1] 27.00923
```

```
(res.df <- df.residual(DL.FM))
```

```
## [1] 39
```

```r
1-pchisq(res.dev, res.df)
```

```
## [1] 0.9266196
```

```r
# No indication of overdispersion: residual deviance is even less than df.
# We continue with the binomial GLM.
```

Lemuricola: continued assumption checking

Now the full model is fitted, for which we check assumptions.

```r
DL.FM  <- glm(cbind(k.l, n-k.l) ~ Species + Dist + Season,
              contrasts=list(Species=contr.sum, Dist=contr.sum, Season=contr.sum),
              family=binomial, data=Qdist.grp)

par(mfrow=c(2,2), mar=c(3,3,2,0))
plot(DL.FM)
```

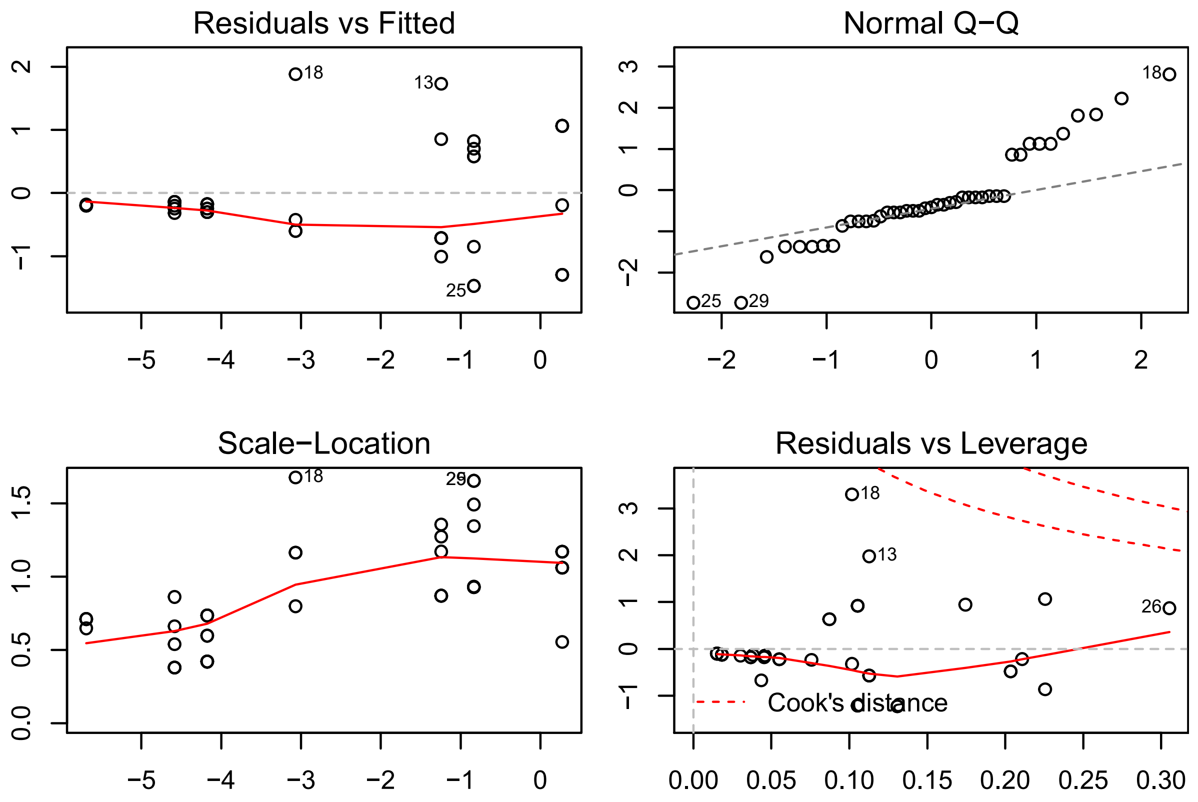


```r
par(mfrow=c(1,1))

# No outliers are found.
# Some observations have rather large (negative) linear predictor values.
sel.obs <- (predict(DL.FM) < -4)
data.frame(Qdist.grp[sel.obs,], predict(DL.FM)[sel.obs])
```

```
##     Season SpLoc Dist     Species Group k.c k.l n          w dSp dD dS dDS
```

```
## 14    Dry  Valo    N    Rufifrons    55    1    0 1 1.0000000   1   1 -1   -1
## 15    Dry  Valo    N    Rufifrons    64    5    0 5 0.3488406   1   1 -1   -1
## 16    Dry  Valo    N    Rufifrons    65    1    0 1 1.0000000   1   1 -1   -1
## 17    Dry  Valo    N    Rufifrons    66    2    0 2 0.6818214   1   1 -1   -1
## 22    Dry  Vato    N    Rufifrons    62    3    0 3 0.5172451   1   1 -1   -1
## 23    Dry  Vato    N    Rufifrons    63    0    0 1 1.0000000   1   1 -1   -1
## 31    Wet  Valo    N Rubriventer    40    1    0 1 1.0000000  -1   1  1    1
## 32    Wet  Valo    N Rubriventer    79    2    0 2 0.6818214  -1   1  1    1
## 33    Wet  Valo    N Rubriventer    81    3    0 3 0.5172451  -1   1  1    1
## 34    Wet  Vato    N Rubriventer    11    0    0 1 1.0000000  -1   1  1    1
## 35    Wet  Vato    N Rubriventer    25    3    0 3 0.5172451  -1   1  1    1
## 36    Wet  Vato    N Rubriventer    82    1    0 1 1.0000000  -1   1  1    1
## 37    Wet  Vato    N Rubriventer    83    1    0 3 0.5172451  -1   1  1    1
## 38    Wet  Vato    N Rubriventer    84    1    0 1 1.0000000  -1   1  1    1
## 39    Wet  Vato    N Rubriventer    85    1    0 2 0.6818214  -1   1  1    1
## 40    Wet  Vato    N    Rufifrons    30    2    0 6 0.3000031   1   1  1    1
## 41    Wet  Vato    N    Rufifrons    32    2    0 6 0.3000031   1   1  1    1
## 42    Wet  Vato    N    Rufifrons    34    5    0 5 0.3488406   1   1  1    1
## 43    Wet  Vato    N    Rufifrons    35    6    0 6 0.3000031   1   1  1    1
##    predict.DL.FM..sel.obs.
## 14               -4.584178
## 15               -4.584178
## 16               -4.584178
## 17               -4.584178
## 22               -4.584178
## 23               -4.584178
## 31               -4.174355
## 32               -4.174355
## 33               -4.174355
## 34               -4.174355
## 35               -4.174355
## 36               -4.174355
## 37               -4.174355
## 38               -4.174355
## 39               -4.174355
## 40               -5.691024
## 41               -5.691024
## 42               -5.691024
## 43               -5.691024
```

```
# These observations are from the less disturbed area, where hardly any
# Lemuricola infection was found. In logistic regression predicted values on
# logist scale will tend towards minus infinity.

# Check GLM leverages.
L.lev <- hatvalues(DL.FM)
range(L.lev)
```

```
## [1] 0.01514451 0.30526202
```

```r
(cutoff.lev <- 2*5/nrow(Qdist.grp))
```

```
## [1] 0.2325581
```

```r
sum(L.lev > cutoff.lev)
```

```
## [1] 1
```

```r
# Largest leverage is 0.31, larger than the cutoff 0.23 (=2*(5/43)).
# 1 observation has leverage exceeding the cutoff.
Qdist.grp[L.lev > cutoff.lev,]
```

```
##    Season SpLoc Dist     Species Group k.c k.l n         w dSp dD dS dDS
## 26    Wet  Tala    Y Rubriventer     5   2   3 7 0.2631608  -1 -1  1  -1
```

```r
# This is the observation with binomial total n=7;
# it indeed should have larg(er) influence...

# Collinearity of explanatory variables: variance inflation factors.
vif(DL.FM)
```

```
##  Species     Dist   Season
## 1.293037 1.025088 1.321446
```

```r
# Largest VIF = 1.32 for Season, which is small. Collinearity is no problem here.

# Although all leverages are rather small, we check DFbeta's:
# Although all leverages are rather small, we check DFbetas:
L.dfbetas <- dfbetas(DL.FM)
par(mfrow=c(1,4), mar=c(4,3,2,0))
hist(L.dfbetas[,1], main="overall mean", xlab="dfbetas")
hist(L.dfbetas[,2], main="half species diff", xlab="dfbetas")
hist(L.dfbetas[,3], main="half disturbance diff", xlab="dfbetas")
hist(L.dfbetas[,4], main="half season diff", xlab="dfbetas")
```

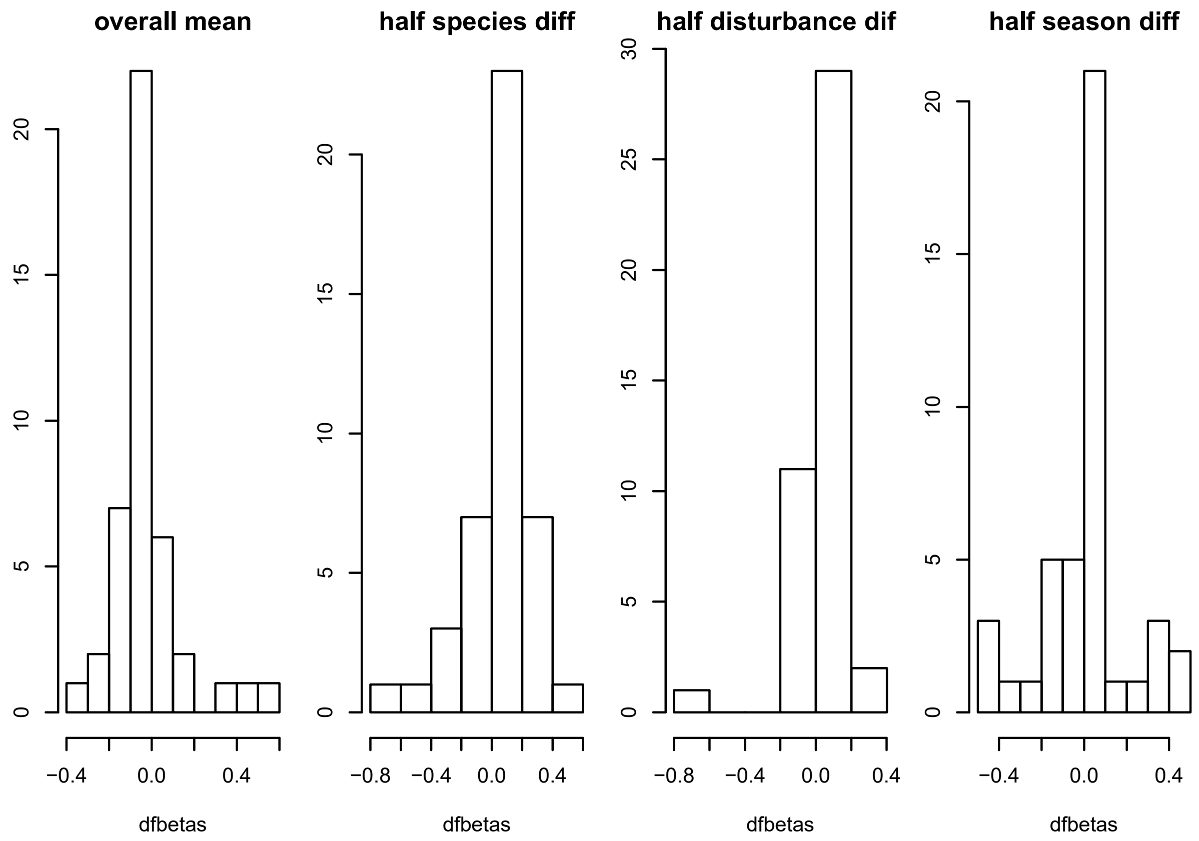


```
par(mfrow=c(1,1))
# No dfbeta comes close to value 2. It appears there are no
# observations with unduely large influence on individual parameters

# Finally, an influencePlot, plotting studentized residuals versus
# leverage with bubble size defined by Cook's distance.
influencePlot(DL.FM)
```

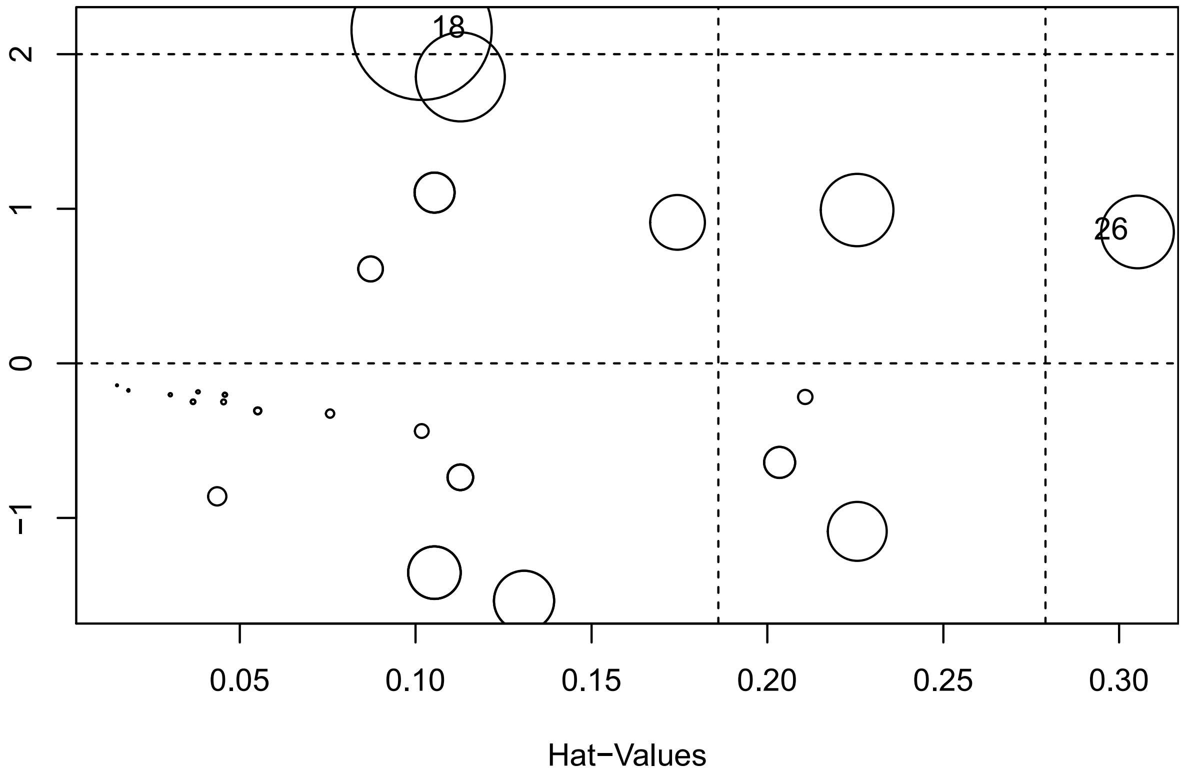


```
##       StudRes       Hat      CookD
## 18 2.1565422 0.1017405 0.30790052
## 26 0.8493375 0.3052620 0.08270219
```

```
# Observation 18 seems to be have a large influence: relatively large
# studentized residual (around 2) combined with large Cook's distance.
Qdist.grp[18,]
```

```
##    Season SpLoc Dist     Species Group k.c k.l n         w dSp dD dS dDS
## 18     Dry  Vato    N Rubriventer    49   2   1 2 0.6818214  -1  1 -1  -1
```

```
# This is the single observation in the undisturbed site with Lemuricola infection.
# If it would not have been there, the contrast will become even bigger.

# Our conclusion is that, although some observations are more influential than others,
# no observation has an overwhelmingly large impact.
```

Lemuricola: model comparisons of Full Model with submodels

We now perform model comparisons, always comparing with the full model.

```
DL.FM  <- glm(cbind(k.l, n-k.l) ~ Species + Dist + Season,
              contrasts=list(Species=contr.sum, Dist=contr.sum, Season=contr.sum),
              family=binomial, data=Qdist.grp)
logLik(DL.FM)
```

```
## 'log Lik.' -19.17619 (df=4)
```

```
# Null model: intercept only, no fixed effects
DL.null  <- glm(cbind(k.l, n-k.l) ~ 1,  family=binomial, data=Qdist.grp)
logLik(DL.null)
```

```
## 'log Lik.' -32.1288 (df=1)
```

```
anova(DL.FM, DL.null, test="Chisq")
```

```
## Analysis of Deviance Table
##
## Model 1: cbind(k.l, n - k.l) ~ Species + Dist + Season
## Model 2: cbind(k.l, n - k.l) ~ 1
##   Resid. Df Resid. Dev Df Deviance  Pr(>Chi)
## 1        39     27.009
## 2        42     52.914 -3  -25.905 9.983e-06
```

```
# Highly significant model comparison: fixed effects are found.

drop1(DL.FM, test="Chisq")
```

```
## Single term deletions
##
## Model:
## cbind(k.l, n - k.l) ~ Species + Dist + Season
##         Df Deviance    AIC     LRT  Pr(>Chi)
## <none>       27.009 46.352
## Species  1   29.630 46.973  2.6209    0.1055
## Dist     1   45.447 62.790 18.4381 1.755e-05
## Season   1   29.132 46.475  2.1224    0.1452
```

```
# Highly significant effect of disturbance only.

summary(DL.FM)
```

```
##
## Call:
## glm(formula = cbind(k.l, n - k.l) ~ Species + Dist + Season,
##     family = binomial, data = Qdist.grp, contrasts = list(Species = contr.sum,
##         Dist = contr.sum, Season = contr.sum))
##
## Deviance Residuals:
##     Min       1Q   Median       3Q      Max
## -1.4715  -0.6032  -0.2011  -0.1426   1.8826
##
## Coefficients:
##             Estimate Std. Error z value Pr(>|z|)
## (Intercept)  -2.7087     0.6252  -4.333 1.47e-05
## Species1      0.7583     0.4962   1.528  0.12644
## Dist1         1.6705     0.5441   3.070  0.00214
## Season1       0.5534     0.3862   1.433  0.15181
##
## (Dispersion parameter for binomial family taken to be 1)
```

```
##
##     Null deviance: 52.914  on 42  degrees of freedom
## Residual deviance: 27.009  on 39  degrees of freedom
## AIC: 46.352
##
## Number of Fisher Scoring iterations: 6
```

Lemuricola: mean prevalences by disturbance.

Now the backtransformed predicted means for disturbance.

```
(emm.dist.lem <- summary(emmeans(DL.FM,"Dist")))
```

```
##  Dist    emmean        SE  df asymp.LCL  asymp.UCL
##  Y    -1.038174 0.4631542 Inf -1.945940 -0.1304085
##  N    -4.379267 1.0766991 Inf -6.489558 -2.2689753
##
## Results are averaged over the levels of: Species, Season
## Results are given on the logit (not the response) scale.
## Confidence level used: 0.95
```

```
emm.dist.lem.back <- 1/(1+exp(-emm.dist.lem[,2]))
emm.dist.lem.lo   <- 1/(1+exp(-emm.dist.lem[,5]))
emm.dist.lem.up   <- 1/(1+exp(-emm.dist.lem[,6]))
data.frame(dist=emm.dist.lem[,1], emm.dist.lem.lo, emm.dist.lem.back, emm.dist.lem.up)
```

```
##   dist emm.dist.lem.lo emm.dist.lem.back emm.dist.lem.up
## 1    Y     0.124996787        0.26150247      0.46744400
## 2    N     0.001516916        0.01237938      0.09372522
```

Lemuricola: Rsquared

```
r.squaredGLMM(DL.FM)
```

```
##       R2m       R2c
## 0.5440784 0.5440784
```

The pseudo R^2_m is 54%. There are no random effects in this glm.

Figure S1 *Callistoura* spp. prevalence as explanatory variable of the faecal microbiota composition. dbRDA Analyses of the abundance-weighted phylogenetic composition of faecal microbiota at OTU level of individual lemurs with *Callistoura* spp. prevalence as explanatory variable. Each plot is based on a subset of the samples defined by season, habitation area and lemur species, as is specified above the plot. The percentages of variation captured by the first two ordination axes is given in the plots. No statistically significant grouping (ANOVA) by *Callistoura* spp. prevalence was observed for any of the subsets.

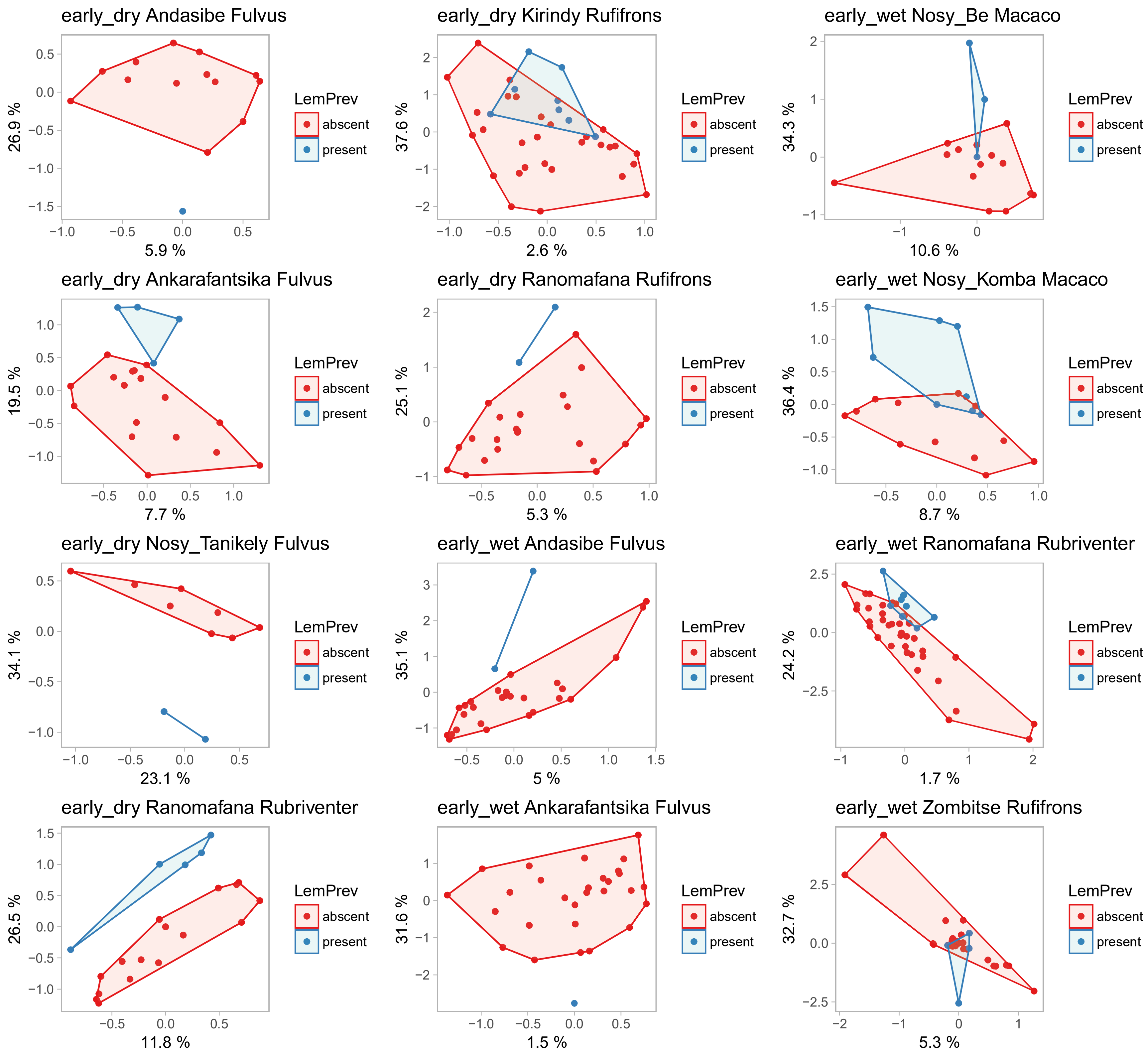
early_dry Andasibe Fulvus
26.9 %
5.9 %
early_dry Kirindy Rufifrons
37.6 %
2.6 %
early_wet Nosy_Be Macaco
34.3 %
10.6 %
early_dry Ankarafantsika Fulvus
19.5 %
7.7 %
early_dry Ranomafana Rufifrons
25.1 %
5.3 %
early_wet Nosy_Komba Macaco
36.4 %
8.7 %
early_dry Nosy_Tanikely Fulvus
34.1 %
23.1 %
early_wet Andasibe Fulvus
35.1 %
5 %
early_wet Ranomafana Rubriventer
24.2 %
1.7 %
early_dry Ranomafana Rubriventer
26.5 %
11.8 %
early_wet Ankarafantsika Fulvus
31.6 %
1.5 %
early_wet Zombitse Rufifrons
32.7 %
5.3 %
LemPrev
abscent
present

Figure S2 *Lemuricola* spp. prevalence as explanatory variable of the faecal microbiota composition. dbRDA Analyses of the abundance-weighted phylogenetic composition of faecal microbiota at OTU level of individual lemurs with *Lemuricola* spp. prevalence as explanatory variable. Each plot is based on a subset of samples as defined by season, habitation area and lemur species, as specified above the plots. The percentage of variation captured by the first two ordination axes is given in the plots. Significant grouping (ANOVA, P= 0.046) by *Lemuricola* spp. prevalence was observed in samples from the *E. rubriventer* population in Ranomafana National Park collected during early dry season, with 9.2% of the observed variation explained by the prevalence of this parasite.

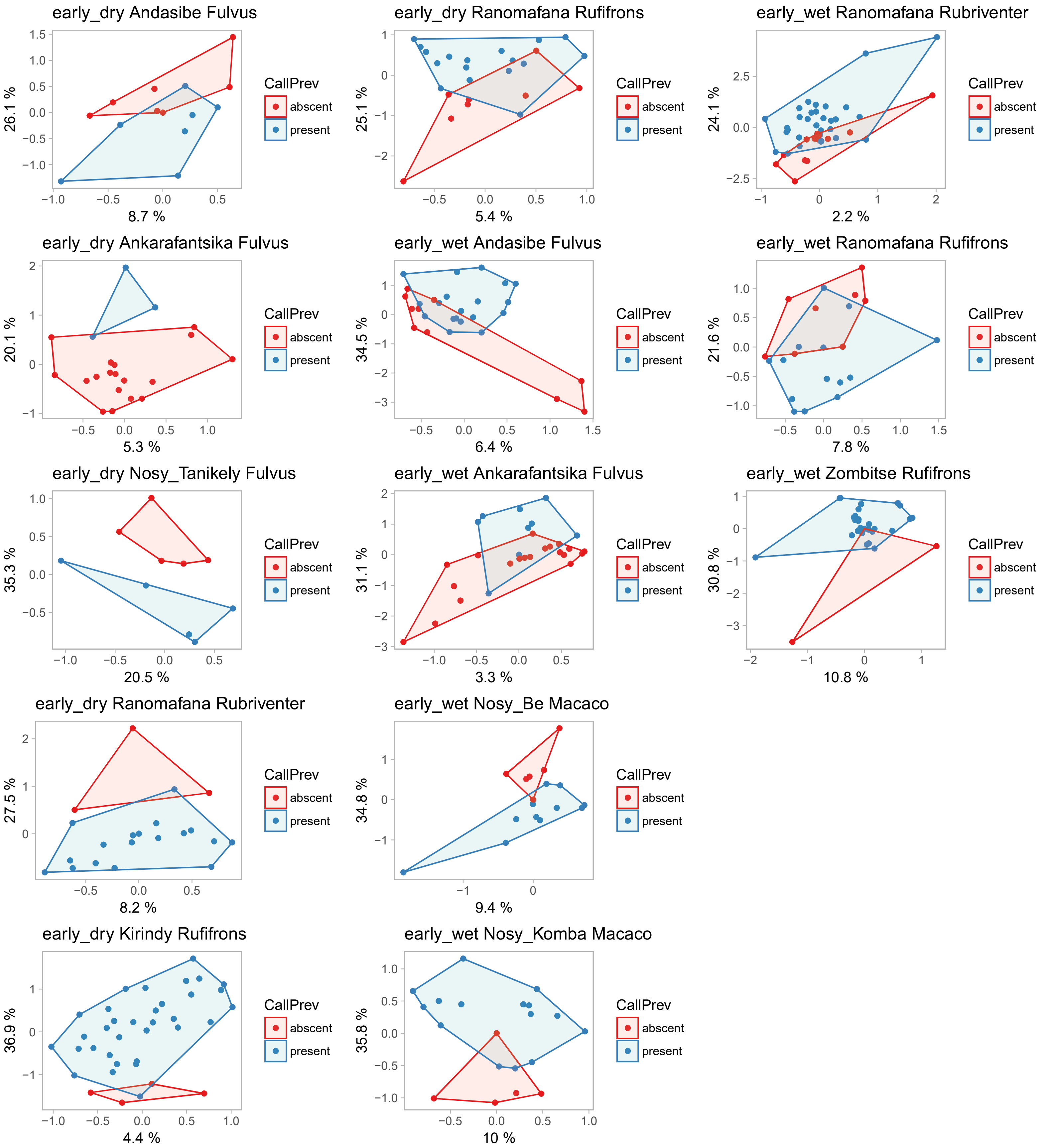
early_dry Andasibe Fulvus
26.1 %
8.7 %
CallPrev
abscent
present
early_dry Ranomafana Rufifrons
25.1 %
5.4 %
CallPrev
abscent
present
early_wet Ranomafana Rubriventer
24.1 %
2.2 %
CallPrev
abscent
present
early_dry Ankarafantsika Fulvus
20.1 %
5.3 %
CallPrev
abscent
present
early_wet Andasibe Fulvus
34.5 %
6.4 %
CallPrev
abscent
present
early_wet Ranomafana Rufifrons
21.6 %
7.8 %
CallPrev
abscent
present
early_dry Nosy_Tanikely Fulvus
35.3 %
20.5 %
CallPrev
abscent
present
early_wet Ankarafantsika Fulvus
31.1 %
3.3 %
CallPrev
abscent
present
early_wet Zombitse Rufifrons
30.8 %
10.8 %
CallPrev
abscent
present
early_dry Ranomafana Rubriventer
27.5 %
8.2 %
CallPrev
abscent
present
early_wet Nosy_Be Macaco
34.8 %
9.4 %
CallPrev
abscent
present
early_dry Kirindy Rufifrons
36.9 %
4.4 %
CallPrev
abscent
present
early_wet Nosy_Komba Macaco
35.8 %
10 %
CallPrev
abscent
present

Supplementary information

Ethanol as a preservation medium

70% Ethanol may perform quite poorly as a preservation medium for studies of the bacterial microbiome, particularly for samples that are not frozen relatively close to the time of collection, as was the case for our samples. Although we are aware of reports that this method of preservation has been shown to affect apparent microbiota profiles, and have therefore also adjusted our collection method in more recent studies, there is also contradicting reports, showing that storage in 70% ethanol safeguards preservation of profiles observed in fresh samples, such as shown for canine fecal samples (Horng et al. 2018). Also, while we did not record this specifically, potential differences in sample collection with respect to time between sampling and preservation were not different for different sampling campaigns; it can probably be excluded that storage significantly affected our observations.

Horng KR, Ganz HH, Eisen JA, Marks SL (2018). Effects of preservation method on canine (Canis lupus familiaris) fecal microbiota. PeerJ 6: e4827.

Additional statistical analyses for revision
Following the reviewer's suggestion, we revised the model used for construction of distance base RDA. The following model had been used in the analysis:

dist.wu ~ Site + Fieldseason + Species + Sex + Age + LemPrev + CallPrev.

The grouping by Callistara prevalence remained significant (p=0.03).

In addition, we used also here an alternative model where co-variables were partialled out with the following model:

dist.wu ~ CallPrev + Condition(Site + Fieldseason + Species + Sex + Age + LemPrev)). This approach also showed significance of Callistara prevalence in explaining the observed variation in microbiota composition (p=0.018)

Prepare environment and load data

```
library(phyloseq)
library(vegan)
library(foreach)
library(doParallel)
library(ggplot2)


#Register a clustor
cl <- makeCluster(68)
registerDoParallel(cl)


set.seed(5626348)

phylo.season <- readRDS("LemII_phylo_season.RDS")

dist.wu <- phyloseq::distance(phylo.season, "wunifrac")
meta.phylo.season <- as(sample_data(phylo.season), "data.frame")
```

1. Make dbRDA model with all variables

1.1 The variable CallPrev at the end

In [17]:

```
dbrda.all <- dbrda(dist.wu ~ Site + Species + Fieldseason + Sex + Age +  LemPrev + CallPrev, data=meta
.phylo.season)
```

```
dbrda.anova <- anova.cca(dbrda.all, by="terms", parallel = 7, permutations = 9999)
dbrda.anova
```

	Df	SumOfSqs	F	Pr(>F)
Site	7	9.15900114	13.8850389	0.0001
Species	1	0.70530710	7.4847153	0.0001
Fieldseason	1	2.73409021	29.0141511	0.0001
Sex	2	0.39843219	2.1140802	0.0011
Age	2	0.34843603	1.8488007	0.0052
LemPrev	1	0.09091047	0.9647414	0.4638
CallPrev	1	0.16953189	1.7990716	0.0307
Residual	308	29.02376095	NA	NA

1.2 Variables other than CallPrev are partialled out

In [13]:

```
dbrda.all.cond <- dbrda(dist.wu ~ CallPrev + Condition(Site + Species + Fieldseason + Sex + Age + Lem
Prev), data=meta.phylo.season)


dbrda.anova.cond <- anova.cca(dbrda.all.cond, by="terms", permutations = 9999)
dbrda.anova.cond
```

	Df	SumOfSqs	F	Pr(>F)
CallPrev	1	0.1695319	1.799072	0.0185
Residual	308	29.0237609	NA	NA

2. In-silico experiment

We performed an in-silico experiment to test the chance of statistically significant sample grouping when *Callistara* prevalence would be assigned randomly. We have generated 1000 datasets where *Callistara* prevalence is randomly shuffled, while the proportion of negative to positive samples is retained. Distance base RDA construction and testing of significance were performed as described in the main text using the full model (dist.wu ~ Site + Fieldseason + Species + Sex + Age + LemPrev + CallPrev). We also used an alternative method for model construction where variables other than CallPrev were partialled out as co-variables (dist.wu ~ CallPrev + Condition(Site + Fieldseason + Species + Sex + Age + LemPrev)).

Out of 1000 alterations, 35 (3.5%) showed P-values ≤ 0.05, and 14 (1.4%) ≤ 0.025 when the full modewas used. When we used the other method where co-variables were partialled out, we observed that 52 (5.2%) alterations showed a P-value ≤ 0.05, and 27 (2.7%) ≤ 0.025.

The data on Callistara prevalence will be randomly shuffled and tested for significance:

# Prepare data:

```
meta.d.random <- meta.phylo.season
 CallPrev.r.mat <- c()
 CallPrev.r.meta.l <- list()

 for (i in 1:1000) {
      CallPrev.rand <- sample(as.character(meta.phylo.season$CallPrev))
      CallPrev.r.mat <- cbind(CallPrev.r.mat, CallPrev.rand)
      meta.d.random$CallPrev <- CallPrev.rand
      CallPrev.r.meta.l[[i]] <- meta.d.random
      }
```

# Approach A. *Variables other than CallPrev are partialled out*

```
r.rda.cond <- foreach (i=1:1000, .packages = "vegan") %dopar%  {
          dbrda.r <- dbrda(dist.wu ~ CallPrev +
                    Condition(Site + Fieldseason + Species + Sex + Age + LemPrev ),
                    data=CallPrev.r.meta.l[[i]])
                    anova.cca(dbrda.r, by="terms", permutations = 9999) }
```

*# **Approach B.** The CallPrev at the end of formula*

```
r.rda.end <- foreach (i=1:1000, .packages = "vegan") %dopar%  {
          dbrda.r <- dbrda(dist.wu ~ Site + Fieldseason + Species + Sex +
                                        Age + LemPrev + CallPrev,
                                data=CallPrev.r.meta.l[[i]])
                      anova.cca(dbrda.r, by="terms", permutations = 9999) }
```

# Extract data from list of anova.cca objects

```
#Function to extract data from list of anova.cca objects
#Will return a matrix with Pval, Sum of squeres and Degrees of freedom


extr_data_anovaCCA_list <- function (anovaCCA_list)  {

   out <- c()

   for(i in 1:length(anovaCCA_list)) {
      anovaCCA <- anovaCCA_list[[i]]
      out <- rbind(out, anovaCCA)}

      colnames(out) <- c("Df", "SumOfSqs", "F", "Pval")
   return(out)
   }
```

```
r.rda.cond.summary <- extr_data_anovaCCA_list(r.rda.cond)
r.rda.end.summary <- extr_data_anovaCCA_list(r.rda.end)
```

2.1 Number of alterations where results are statistically significant

Where P-values are below 0.05 and 0.025, respectively

# a) For the model with the conditional statement

```
r.rda.cond.f <- r.rda.cond.summary[grepl("CallPrev", rownames(r.rda.cond.summary)), ]
table(r.rda.cond.f$Pval <= 0.05)
table(r.rda.cond.f$Pval <= 0.025)
```

```
FALSE  TRUE
  948    52

FALSE  TRUE
  973    27
```

# b) For the model where CallPrev at the end

```
r.rda.end.f <- r.rda.end.summary[grepl("CallPrev", rownames(r.rda.end.summary)), ]
table(r.rda.end.f$Pval <= 0.05)
table(r.rda.end.f$Pval <= 0.025)
```

```
FALSE  TRUE
  965    35

FALSE  TRUE
  986    14
```
